# Ultrasound‐Triggered piRNA Delivery Using Biomimetic Phase‐Changeable Nanoparticles for Theranostics of Myocardial Infarction

**DOI:** 10.1002/advs.78282

**Published:** 2026-10-14

**Authors:** Yajun Zhang, Hongzhao Qi, Yanyan Yang, Feng Wang, Tiantian Han, Yanbei Lei, Xiaolu Li, Zhibin Wang, Hua Qu, Tao Yu, Pin Sun

**Affiliations:** ^1^ Department of Cardiac Ultrasound The Affiliated Hospital of Qingdao University Qingdao Shandong People's Republic of China; ^2^ Department of Vascular Biology and Translational Medicine Institute for Translational Medicine The Affiliated Hospital of Qingdao University Qingdao Shandong People's Republic of China; ^3^ Department of Immunology School of Basic Medicine Qingdao University Qingdao Shandong People's Republic of China; ^4^ Organ Transplantation Center The Affiliated Hospital of Qingdao University Qingdao Shandong People's Republic of China; ^5^ Department of Ophthalmology The Affiliated Hospital of Qingdao University Qingdao Shandong People's Republic of China; ^6^ Institute of Clinical Pharmacology Xiyuan Hospital China Academy of Chinese Medical Sciences Beijing People's Republic of China

**Keywords:** cardiomyocyte membrane, myocardial infarction, necroptosis, piRNAs, ultrasound‐responsive nanoparticles

## Abstract

Myocardial infarction (MI) remains a major cause of cardiovascular morbidity, and effective strategies to prevent adverse remodeling are limited. Here, we identify piR‐1873 as a cardioprotective piRNA that is down‐regulated in MI patients, mouse models, and H_2_O_2_‐treated cardiomyocytes. Serum piR‐1873 levels correlated with MI biomarkers, suggesting its potential as a complementary biomarker, with an area under the curve (AUC) of 0.8389. Mechanistically, piR‐1873 directly targeted Usp2 and inhibited RIP1/RIP3/MLKL‐mediated necroptosis. To achieve efficient delivery, we developed ultrasound (US)‐responsive piRNA nanoparticles (piR‐NPs) consisting of a perfluorohexane (PFH) phase‐change core loaded with piR‐1873 and coated with a cardiomyocyte membrane shell. In vitro, piR‐NPs enhanced piR‐1873 delivery and suppressed cardiomyocyte necroptosis, with US further improving these effects. In MI mice, piR‐NPs accumulated in infarcted myocardium, and US enhanced nanoparticle retention, resulting in improved cardiac function, reduced fibrosis, and attenuated necroptosis without detectable systemic toxicity. Collectively, this study reveals the diagnostic and therapeutic potential of piR‐1873 and establishes a biomimetic, US‐triggered piRNA delivery platform for MI treatment.

## Graphical abstract

A biomimetic ultrasound‐responsive piRNA delivery nanoparticle platform (piR‐NPs) enables targeted delivery and ultrasound (US)‐triggered release of piR‐1873 in infarcted myocardium. The released piR‐1873 suppresses Usp2‐mediated RIP1/RIP3/MLKL necroptosis, reduces myocardial injury and fibrosis, and promotes cardiac functional recovery, providing a promising strategy for precision therapy of myocardial infarction (MI). The graphical abstract was created using Adobe Illustrator.

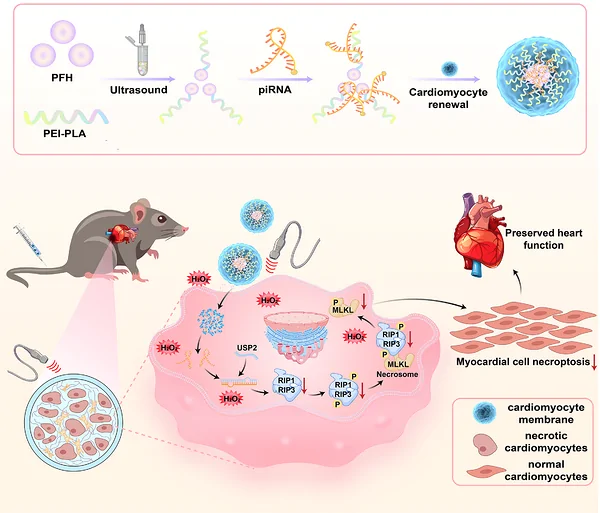

## 1 Introduction

Myocardial infarction (MI) is one of the major causes of morbidity and mortality worldwide [1], and its pathological mechanism is myocardial ischemic necrosis caused by acute coronary artery occlusion [2]. Percutaneous coronary intervention (PCI) and medical therapies can effectively restore perfusion and improve short‐term prognosis [3], but they cannot prevent adverse ventricular remodeling, progressive myocardial fibrosis, and the eventual progression to heart failure [4]. The current treatment is mainly to control symptoms and promote angiogenesis, but there is still a lack of effective methods and targets to precisely regulate post‐infarction repair and carry out targeted intervention. Therefore, developing a highly efficient, controllable, and infarct‐microenvironment‐targeted therapeutic platform is of great significance for improving the long‐term prognosis of MI.

Ventricular remodeling after MI is a multifactorial process. Studies have emphasized that non‐coding RNAs (ncRNAs) play a critical role in cardiovascular diseases [5, 6]. PIWI‐interacting RNAs (piRNAs), a class of small RNAs ranging from 24 to 31 nucleotides in length and characterized by 3’‐terminal 2’‐O‐methylation modifications, are uniformly expressed in somatic tissues [7, 8]. This unique 3’‐end modification confers exceptional resistance to nuclease degradation, endowing piRNAs with stability that makes them attractive candidates for therapeutic intervention. Research has shown that piRNAs, acting as transcriptional and post‐transcriptional regulators, are significantly changed under pathological conditions such as cardiac hypertrophy, heart failure, and ischemia‐reperfusion injury [9, 10, 11, 12], and piRNAs may be involved in the progression of heart diseases by regulating cell apoptosis, inflammation, and fibrosis [13, 14, 15, 16]. Previous studies have demonstrated that the expression profile of specific piRNAs changed significantly after MI [17], suggesting their potential involvement in myocardial injury and repair. However, the dynamic expression patterns, functional mechanisms, and downstream signaling pathways remain poorly understood.

Furthermore, even if specific piRNAs with cardioprotective potential are identified, their in vivo delivery still faces physiological challenges, such as nuclease‐mediated degradation, renal clearance, insufficient tissue specificity, and difficulties in crossing vascular endothelium and cell membranes [7]. In recent years, biomimetic nanotechnology of cell membranes has emerged as a promising approach to enhance targeting specificity [18, 19]. By wrapping the cell membrane derived from cardiomyocytes on nanocarriers, they can be made to have the surface antigen characteristics of cells and promote their active targeting of damaged myocardium [20]. This approach significantly enhances the accumulation of the carriers in cardiac tissue. However, cardiomyocyte membrane coating primarily solves the targeting issue alone. The pathological microenvironment of the infarction area is complex, and tissue edema, microcirculation dysfunction, and extracellular matrix (ECM) remodeling will severely limit the subsequent penetration of nanoparticles into deeper ischemic myocardium and effective delivery into cells [21]. Therefore, homogeneous drug distribution and efficient intracellular release cannot be achieved by a simple biomimetic targeting strategy.

To overcome these delivery barriers, this study developed a theranostic nanodelivery system that combines active targeting of cardiomyocyte‐derived membranes with ultrasound (US) responsiveness. The system consists of a phase‐changeable liquid fluorocarbon core, perfluorohexane (PFH), loaded with a therapeutic piRNA, piR‐1873, and encapsulated within a cardiomyocyte membrane shell. This design confers multiple functions: (i) the cardiomyocyte membrane shell actively targets the injured myocardium; (ii) the perfluorohexane core responds to US by undergoing a phase transition, generating enhanced contrast signals that enable real‐time monitoring of the delivery process; and (iii) US exposure induces inertial cavitation, which transiently permeabilizes the vascular endothelium and cell membranes, thereby enhancing local tissue permeability and intracellular drug delivery for spatiotemporally controlled release [22, 23, 24]. Using this platform, we systematically evaluated the cardioprotective effects of piR‐1873 delivery on cardiac structure and function in MI models. Mechanistically, we identified that piR‐1873 directly targets Usp2 and suppresses the RIP1/RIP3/MLKL‐mediated necroptosis pathway in cardiomyocytes. By defining this specific piR‐1873‑Usp2‑necroptosis axis in post‑infarction ventricular remodeling, our study fills the current knowledge gap in piRNA‐mediated cardiac repair and assesses the potential of piRNAs as actionable therapeutic targets. These mechanistic insights not only advance our fundamental understanding of piRNA biology in the ischemic heart, but also validate a targeted, US‐responsive RNA delivery strategy, thereby providing both a theoretical foundation and a technical framework for precision theranostics in MI.

## 2 Results

### 2.1 PiR‐1873 Is Down‐Regulated in MI and Correlates with Cardiac Function

To identify the critical piRNAs associated with MI, candidate piRNAs were first validated in both animal and cellular models. We induced MI by permanent ligation of the left anterior descending (LAD) coronary artery in C57BL/6 mice. At 28 days after surgery, MI mice exhibited marked cardiac dysfunction and myocardial fibrosis, confirming the successful establishment of the model (Figure S1A–C). We found that piR‐324, piR‐1872, and piR‐1873 were down‐regulated in the heart tissue of MI mice compared with sham mice, as demonstrated by reverse transcription quantitative polymerase chain reaction (RT‐qPCR) analysis, which was consistent with the sequencing results (Figure 1A; Figure S1D). We next examined the expression of these piRNAs in H_2_O_2_‐treated cardiomyocytes. Similarly, the expression of piR‐324, piR‐1872, and piR‐1873 was down‐regulated, among which piR‐1873 showed the most pronounced reduction (Figure 1B; Figure S1E). Therefore, piR‐1873 was selected as the core candidate molecule for subsequent functional and mechanistic investigations.

**FIGURE 1:**
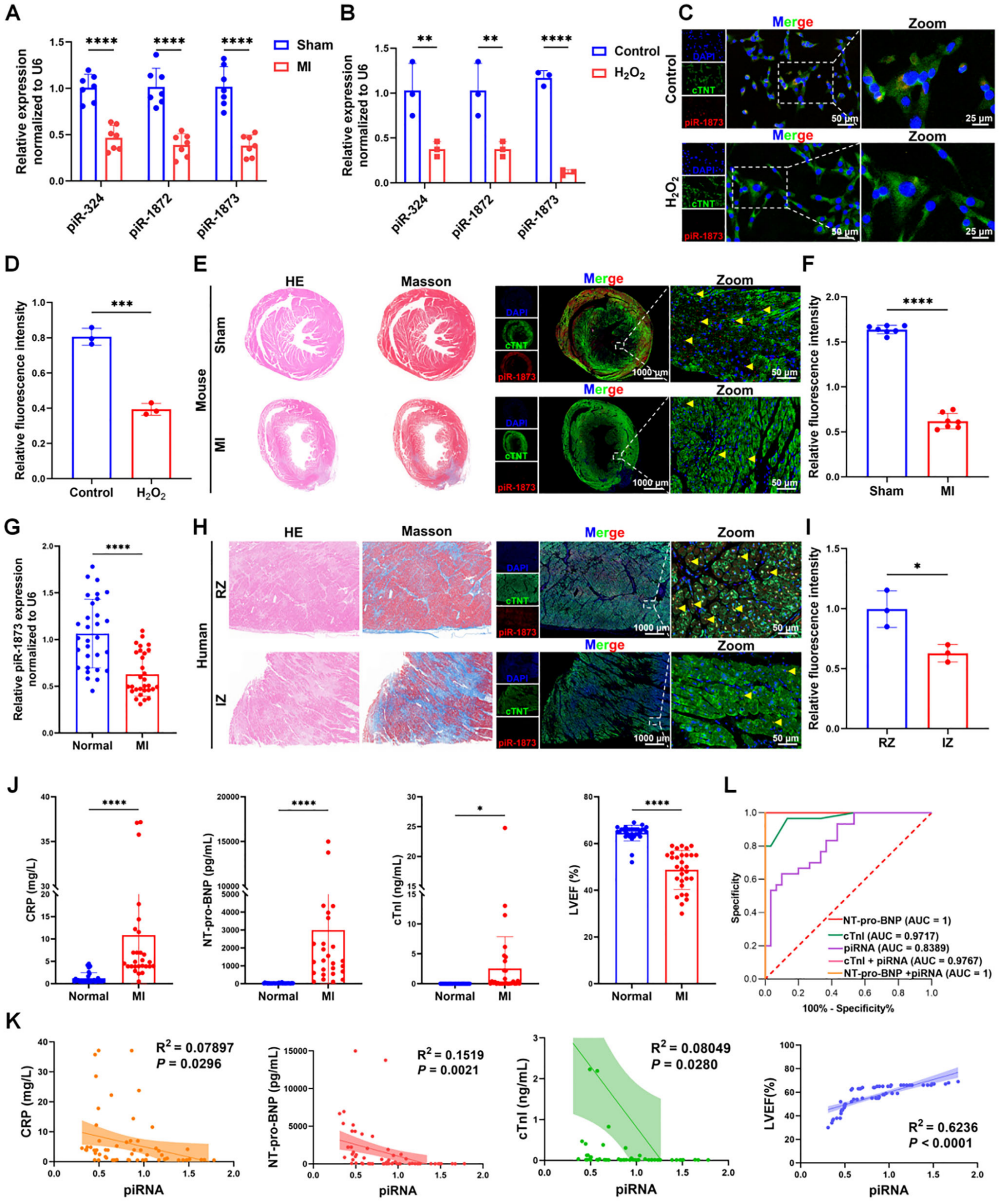
piR‐1873 expression is down‐regulated in MI. (A) Relative expression levels of down‐regulated piRNAs in mouse myocardial tissue were assessed by RT‐qPCR (*n* = 7). (B) Relative expression levels of down‐regulated piRNAs in H_2_O_2_‐treated cardiomyocytes were assessed by RT‐qPCR (*n* = 3). (C, D) FISH combined with immunofluorescence staining was used to detect the expression and subcellular localization of piR‐1873 in the control and H_2_O_2_‐treated cardiomyocytes, and to quantify fluorescence intensity (piR‐1873) (*n* = 3). Scale bars: 50 µm; enlarged scale bar: 25 µm. (E, F) H&E staining, Masson staining, and FISH combined with immunofluorescence staining were used to detect pathological injury and piR‐1873 expression in myocardial tissues from Sham and MI mice, and to quantify fluorescence intensity (piR‐1873) (*n* = 7). Scale bars: 1000 µm; enlarged scale bar: 50 µm. (G) RT‐qPCR was used to detect relative piR‐1873 expression in the serum of patients with MI and healthy controls (Normal) (*n* = 30). (H, I) H&E staining, Masson staining, and FISH combined with immunofluorescence staining were used to detect the pathological changes and piR‐1873 expression in human myocardial tissues from the RZ and IZ, and to quantify fluorescence intensity (piR‐1873) (*n* = 3). Scale bars: 1000 µm; enlarged scale bar: 50 µm. (J) Serum levels of MI‐related clinical indicators, including CRP, NT‐proBNP, cTnI, and LVEF measured by echocardiography, were used to detect MI in patients with MI and healthy controls (Normal) (*n* = 30). (K) Correlation analysis showed that serum piR‐1873 levels were negatively correlated with CRP, NT‐proBNP, and cTnI, but positively correlated with LVEF. (L) ROC curve analysis evaluating the diagnostic performance of piR‐1873, MI‐associated clinical indicators, and piR‐1873 combined with clinical indicators for MI discrimination. ^*^ *p* < 0.05, ^**^ *p* < 0.01, ^***^ *p* < 0.001, ^****^ *p* < 0.0001 compared with indicated groups.

Subsequently, to further explore the expression changes and cellular localization of piR‐1873 in myocardial tissue, hematoxylin‐eosin (H&E), Masson, and fluorescence in situ hybridization (FISH) combined with immunofluorescence staining were conducted. We found that piR‐1873 signals were widely distributed throughout the myocardial tissue and showed enrichment in regions with preserved cardiomyocyte structures. Compared with the Sham group, piR‐1873 expression was markedly lower in infarcted myocardium from MI mice, and the fluorescence intensity was about half that of the Sham group (Figure 1E,F). Through RT‐qPCR analysis, we found that the expression level of piR‐1873 was twofold higher in mouse primary neonatal ventricular cardiomyocytes (NVCMs) than in primary neonatal cardiac fibroblasts (NCFs) (Figure S2A). H_2_O_2_ and hypoxic treatment are commonly used to simulate cellular oxidative stress and ischemia/hypoxia‐induced cellular injury. Compared with H_2_O_2_ treatment, hypoxic stimulation did not significantly alter piR‐1873 expression (Figure S2B). Therefore, H_2_O_2_‐treated mouse cardiomyocytes were used as the cellular injury model for subsequent experiments. To determine the optimal pathological stimulation concentration and duration, HL‐1 cells were exposed to different concentrations of H_2_O_2_ for different treatment times and then analyzed by PI staining. The results showed that treatment with 600 µm H_2_O_2_ for 8 h was the optimal condition for establishing a cardiomyocyte necrosis model (Figure S2C–F). Under this condition, PI staining showed moderate cell death (approximately 10–30%), while preserving sufficient viable cells for subsequent functional and mechanistic analyses. Cellular FISH analysis similarly revealed that the fluorescence intensity of piR‐1873 under H_2_O_2_ stimulation was significantly reduced, and its subcellular localization did not change significantly (Figure 1C,D).

To explore the expression characteristics and clinical relevance of piR‐1873 in clinical MI, we collected serum samples from healthy controls (Normal) and MI patients, and myocardial tissue samples from the infarct zone (IZ) and remote zone (RZ) of MI patients. piR‐1873 levels in serum and myocardial tissues were examined by RT‐qPCR and histological staining. The physical and biochemical parameters are summarized in Table S1. Serum levels of piR‐1873 were decreased in patients with MI compared with Normal controls (Figure 1G). Myocardial tissues from patients with IZ displayed myofiber disruption, inflammatory cell infiltration, and fibrotic remodeling, whereas the RZ myocardial tissues exhibited relatively preserved architecture (Figure 1H,I). piR‐1873 was spatially co‐localized with the cardiomyocyte marker cardiac troponin T (cTNT), and its fluorescence signal in the infarcted myocardium of IZ was significantly weaker (Figure 1H,I). These results indicate that piR‐1873 is significantly down‐regulated in both myocardial tissue and serum from patients with MI.

Meanwhile, the clinical biochemical indicators related to MI were further analyzed. Patients with MI exhibited markedly increased serum levels of C‐reactive protein (CRP), N‐terminal pro‐B‐type natriuretic peptide (NT‐proBNP), and cardiac troponin I (cTnI), together with significantly reduced left ventricular ejection fraction (LVEF) (Figure 1J). Correlation analysis showed that serum piR‐1873 levels were negatively correlated with CRP, NT‐proBNP, and cTnI, but positively correlated with LVEF (all *p* < 0.05) (Figure 1K), suggesting that reduced piR‐1873 expression is closely associated with the severity of myocardial injury and impaired cardiac function. To further characterize the temporal changes of circulating piR‐1873 during MI progression, we examined serum piR‐1873 expression levels at different time points after LAD ligation in a mouse MI model. The observed temporal profile indicated that piR‐1873 decreased during the early stage of MI, reached the lowest level at 72 h after surgery, and gradually recovered during the subsequent repair phase; however, its expression remained below the baseline level even at 28 days (Figure S3A). Meanwhile, classical myocardial injury biomarkers cTnI and CK‐MB exhibited typical dynamic changes after MI. cTnI reached its peak level at 6 h and subsequently declined, whereas CK‐MB peaked at 12 h and gradually returned to baseline over a prolonged time after MI (Figure S3B,C). Receiver operating characteristic (ROC) curve analysis further revealed that the area under the curve (AUC) of piR‐1873 for diagnosing MI was 0.8389, indicating favorable diagnostic potential (Figure 1L). Furthermore, combined diagnostic models incorporating piR‐1873 with conventional clinical indicators were evaluated. The AUC of the cTnI + piR‐1873 model increased from 0.9717 to 0.9767, while the NT‐proBNP + piR‐1873 model achieved an AUC of 1.000 (Table S2). DeLong's test revealed that the difference between cTnI alone and cTnI combined with piR‐1873 did not reach statistical significance (*p* = 0.442), suggesting that piR‐1873 may provide complementary molecular information alongside conventional myocardial injury biomarkers rather than serve as a replacement for established diagnostic indicators.

Collectively, piR‐1873 was significantly down‐regulated in MI patient serum and myocardial tissue, MI mouse myocardium, and H_2_O_2_‐treated cardiomyocytes, and has potential value as a complementary diagnostic marker for MI.

### 2.2 PiR‐1873 Regulates Cardiomyocyte Necroptosis

In order to clarify the functional role and potential mechanism of piR‐1873 in cardiomyocytes, we initially predicted its potential downstream target genes and related biological pathways. Based on miRanda, RIsearch, TargetScan, and RNAhybrid databases, 1380 potential target mRNAs were identified. Through Kyoto Encyclopedia of Genes and Genomes (KEGG) pathway enrichment analysis, it was found that these target genes were significantly enriched in necroptosis‐related pathways, suggesting that necroptosis may represent a major biological process involved in the regulation of cardiomyocyte injury by piR‐1873 (Figures 2A,B). To evaluate the effect of piR‐1873 on H_2_O_2_‐induced cardiomyocyte necroptosis, a mimic and an inhibitor were used to overexpress or knock down piR‐1873 expression, respectively (Figure S4A,B). We showed that piR‐1873 overexpression reduced the proportion of PI‐positive cells induced by H_2_O_2_ from 25% to 10%, and decreased the expression levels of phosphorylated RIP1 (p‐RIP1), p‐RIP3, and p‐MLKL, biomarkers of necroptosis (Figures 2C‐F). These data suggest that piR‐1873 replenishment inhibits cardiomyocyte necroptosis. Conversely, piR‐1873 inhibition increased the proportion of PI‐positive cells (from 5% to 15%) and up‐regulated the expression levels of necroptosis‐related proteins (Figures 2G‐J), suggesting that loss of endogenous piR‐1873 aggravates cardiomyocyte necroptotic injury.

**FIGURE 2:**
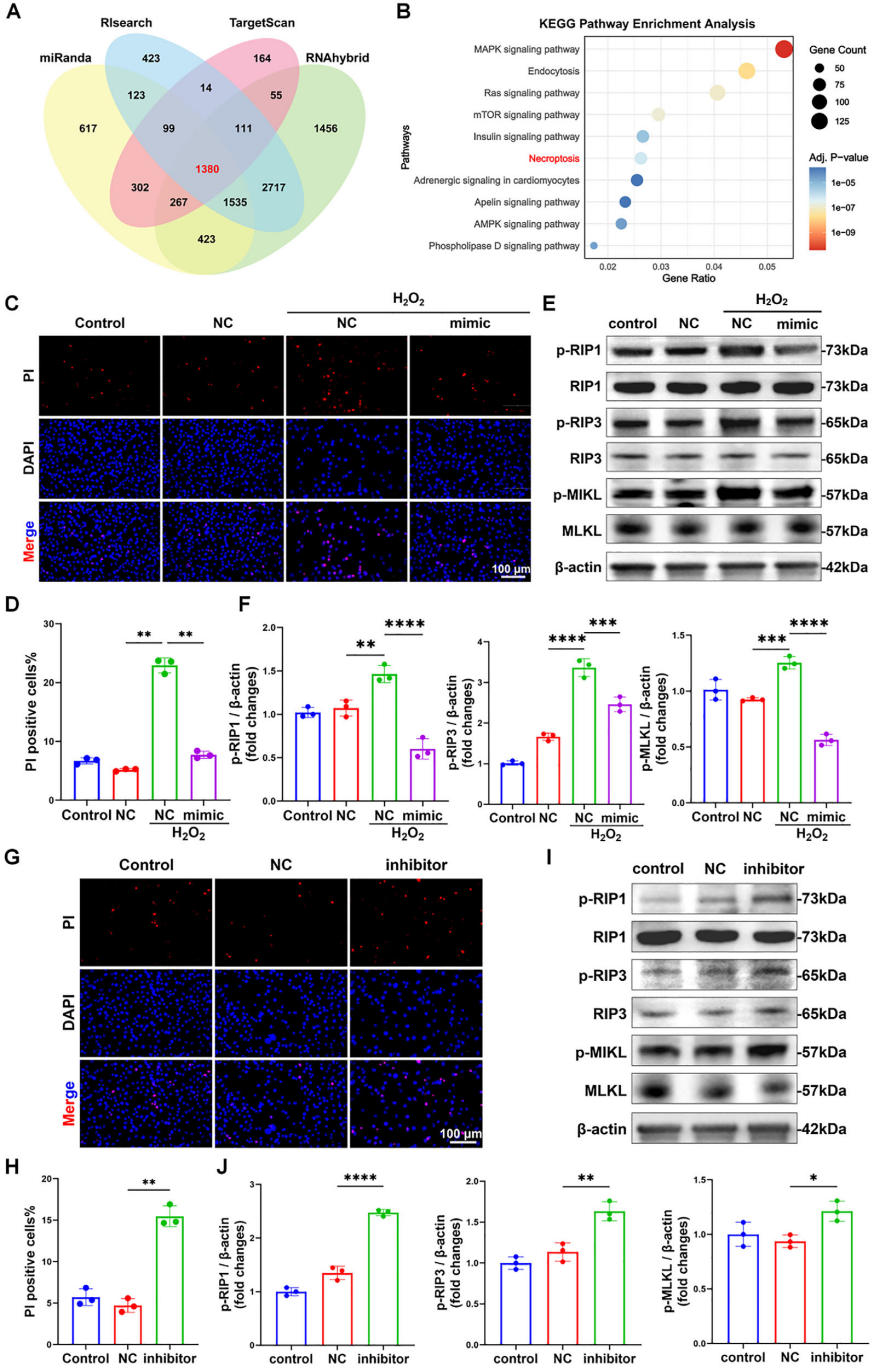
Overexpression of piR‐1873 suppresses H_2_O_2_‐treated cardiomyocytes' necroptosis, whereas knockdown of piR‐1873 promotes necroptosis. (A) Potential target genes of piR‐1873 were predicted by miRanda, RIsearch, TargetScan, and RNAhybrid. (B) KEGG pathway enrichment analysis of piR‐1873 downstream mRNAs. (C, D) PI staining was used to detect cardiomyocyte necroptosis under physiological, H_2_O_2_‐treated, or mimic transfection conditions, and to quantify the proportion of PI‐positive cells (*n* = 3). Scale bars: 100 µm. (E, F) Western blotting was used to detect expression of necroptosis‐related proteins in cardiomyocytes after mimic transfection and quantification (*n* = 3). (G, H) PI staining was used to detect cardiomyocyte necroptosis after inhibitor transfection under physiological conditions, and quantification of the proportion of PI‐positive cells (*n* = 3). Scale bars: 100 µm. (I, J) Western blotting was used to detect expression of necroptosis‐related proteins in cardiomyocytes after inhibitor transfection and quantification (*n* = 3). ^*^ *p* < 0.05, ^**^ *p* < 0.01, ^***^ *p* < 0.001, ^****^ *p* < 0.0001 compared with indicated groups.

Considering that KEGG pathway enrichment also implicated pathways related to cell proliferation and apoptosis, and cardiomyocyte apoptosis is a key event in MI pathogenesis, we further evaluated whether overexpression or knockdown of piR‐1873 affected cardiomyocyte proliferation or apoptosis. Notably, neither overexpression nor knockdown of piR‐1873 significantly affected cardiomyocyte proliferation or apoptosis (Figure S4C–H), indicating that piR‐1873 mainly modulates necroptosis rather than proliferation or classical apoptosis pathways. The RIPK1/RIPK3/MLKL pathway constitutes the core regulatory axis of necroptosis in cardiomyocytes [25, 26]. Therefore, piR‐1873 may participate in MI‐related injury by regulating RIP1/RIP3/MLKL‐associated necroptotic signaling.

### 2.3 PiR‐1873 Directly Targets and Negatively Regulates Usp2

We next explored whether potential downstream targets mediate the protective effect of piR‐1873 on MI. By intersecting the 1380 KEGG‐enriched candidate genes with 115 differentially expressed genes identified by transcriptomic sequencing, we obtained 14 overlapping candidate genes (Figure 3A). Among them, Usp2, Elmo1, Hs3st3a1, Sema3g, Ngfr, Wnt7b, and Atp2a3 have been previously reported to be associated with necroptosis [27, 28, 29, 30, 31], suggesting that these genes may participate in piR‐1873‐mediated regulation of necroptosis. We further verified the interaction between the above genes and piR‐1873 by RNA pull‐down. Among them, Usp2, Hs3st3a1, and Atp2a3 showed markedly greater enrichment with piR‐1873 than the other candidate genes, indicating stronger potential binding affinity with piR‐1873 (Figure 3B). Further validation of Usp2, Hs3st3a1, and Atp2a3 expression after piR‐1873 overexpression or knockdown revealed that Usp2 exhibited the most consistent negative response to piR‐1873. Specifically, piR‐1873 overexpression significantly reduced Usp2 mRNA levels, whereas piR‐1873 inhibition markedly increased Usp2 expression (Figures 3C,D). Usp2 is a deubiquitinating enzyme that has been reported to stabilize RIP1 through deubiquitination, thereby promoting the formation of the RIP1/RIP3 necrosome complex and inducing the phosphorylation and oligomerization of MLKL [32, 33]. Subsequent prediction analysis using the Oebiotech platform (https://cloud.oebiotech.cn/task/detail/array_miranda_plot/), which predicts potential binding sites based on sequence complementarity, successfully identified binding sites between piR‐1873 and the Usp2 3′UTR (Figure 3E). Based on the predicted binding sequences, Usp2 3′UTR wild‐type (WT) and mutant‐type (MUT) reporter plasmids were constructed, followed by dual‐luciferase reporter assays. The results confirmed that piR‐1873 significantly suppressed luciferase activity of the WT Usp2 3′UTR reporter, whereas the MUT reporter was unaffected (Figure 3F). Consistently, Western blot analysis demonstrated that Usp2 protein levels were markedly decreased after piR‐1873 overexpression and increased after piR‐1873 inhibition (Figures 3G‐J), further confirming that Usp2 is negatively regulated by piR‐1873 at the protein level. Taken together, Usp2 might be one of the downstream targets mediating the protective effect of piR‐1873 against MI.

**FIGURE 3:**
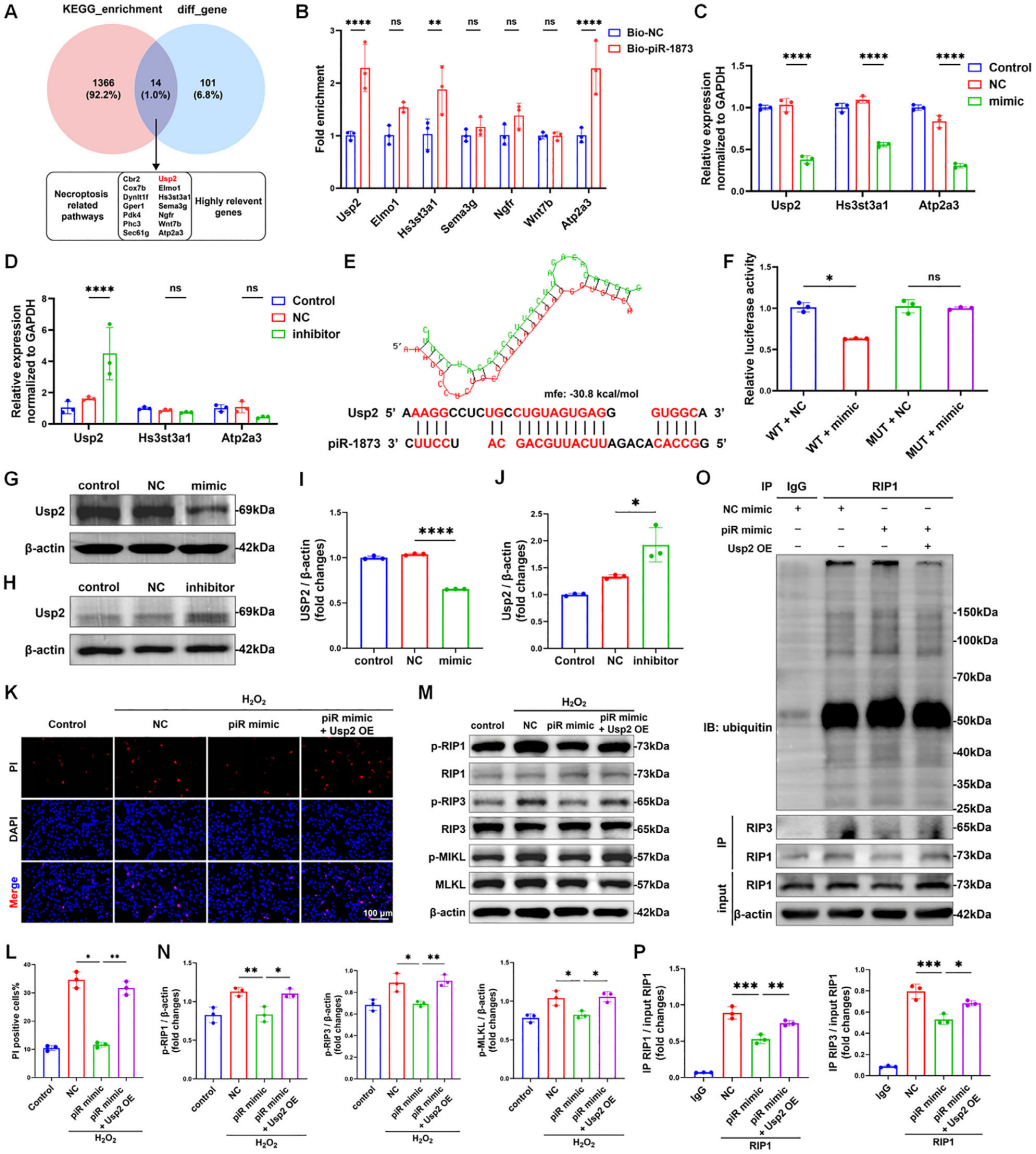
Usp2 is a downstream target of piR‐1873. (A) Venn diagram of intersection analysis between KEGG enrichment analysis results and differentially expressed candidate genes. (B) An RNA pull‐down assay was used to detect binding enrichment of candidate genes with piR‐1873. (C) RT‐qPCR was used to detect mRNA expression levels of Usp2, Hs3st3a1, and Atp2a3 in cardiomyocytes after piR‐1873 overexpression (*n* = 3). (D) RT‐qPCR was used to detect mRNA expression levels of Usp2, Hs3st3a1, and Atp2a3 in cardiomyocytes after piR‐1873 knockdown (*n* = 3). (E) Predicted binding sites of piR‐1873 to USP2 via the Oebiotech website. (F) Dual‐luciferase reporter assay to validate the interaction between piR‐1873 and Usp2 WT or MUT binding sequences (*n* = 3). (G, I) Western blotting was used to detect Usp2 protein expression after piR‐1873 overexpression and quantification (*n* = 3). (H, J) Western blotting was used to detect Usp2 protein expression after piR‐1873 knockdown and quantification (*n* = 3). (K, L) PI staining was performed to evaluate H_2_O_2_‐induced cell death in cardiomyocytes treated with NC mimic, piR‐1873 mimic, or piR‐1873 mimic combined with Usp2 overexpression (Usp2 OE), and the percentage of PI‐positive cells was quantified (*n* = 3). Scale bars: 100 µm. (M, N) Western blot analysis was performed to detect the expression levels of necroptosis‐related proteins under H_2_O_2_ stimulation in different treatment groups, followed by quantitative analysis (*n* = 3). (O, P) Western blot was performed to assess RIP1 ubiquitination levels in cardiomyocytes treated with NC mimic, piR‐1873 mimic, or piR‐1873 mimic combined with Usp2 overexpression. The interaction between RIP1 and RIP3 was further examined to evaluate necrosome formation, followed by quantitative analysis (*n* = 3). ns: no significance, ^*^ *p* < 0.05, ^**^ *p* < 0.01, ^****^ *p* < 0.0001 compared with indicated groups.

To further test the involvement of Usp2 in piR‐1873‐mediated protection against necroptosis, we performed rescue experiments using H_2_O_2_‐treated cardiomyocytes. PI staining and Western blot analyses showed that Usp2 overexpression partially reversed the protective effects of piR‐1873, as shown by increased cardiomyocyte necroptosis (Figure 3K,L) and elevated expression of necroptosis‐associated proteins (Figure 3M,N), indicating that Usp2 serves as an important downstream target involved in piR‐1873‐mediated regulation of cardiomyocyte survival. As Usp2 functions as a deubiquitinating enzyme that regulates RIP1 stability and necrosome formation, we used RIP1 immunoprecipitation assays to examine RIP1 ubiquitination and the interaction between RIP1 and RIP3. piR‐1873 overexpression increased RIP1 ubiquitination and reduced the association between RIP1 and RIP3, whereas Usp2 overexpression abolished these effects (Figure 3O,P). These results demonstrate that piR‐1873 inhibits cardiomyocyte necroptosis by suppressing Usp2, thereby promoting RIP1 ubiquitination and disrupting RIP1/RIP3 necrosome formation. Mechanistically, Usp2 might be one of the downstream targets mediating the protective effect of piR‐1873 against MI, and the piR‐1873/Usp2 axis may contribute to myocardial protection after MI through regulation of RIP1 ubiquitination and necrosome formation.

### 2.4 Preparation and Characterization of piR‐NPs

To achieve efficient delivery of piR‐1873 in vivo, we designed and constructed a biomimetic nanodelivery system consisting of cationic polymeric nanoparticles loaded with piR‐1873, coated with cardiomyocyte membranes, and encapsulating PFH as a US‐responsive core, termed piR‐NPs (Figure 4A). The nucleic acid‐loading capacity of the nanoparticles was first validated by agarose gel electrophoresis. The absence of the piRNA band at an NPs/piRNA mass ratio of 8:1 indicated efficient piRNA encapsulation (Figure 4B). In addition, compared with nanoparticles without cardiomyocyte membrane coating [named as piR‐NPs cell (‐)], cardiomyocyte membrane‐coated piR‐NPs retained nucleic acids effectively and enabled efficient piRNA release after US exposure (Figure 4C). Further nuclease degradation assays showed that piR‐NPs markedly protected piRNA from nuclease‐mediated degradation (Figure 4D). Coomassie blue staining further showed that the protein profile of piR‐NPs was highly consistent with that of cardiomyocyte membrane extracts (Figure 4E), confirming successful membrane coating on the nanoparticle surface and supporting their improved biological stability. To further quantitatively evaluate the delivery performance of piR‐NPs, we systematically analyzed their key compositional parameters. The final piRNA encapsulation efficiency (EE) reached 93.98 ± 0.33%, with a loading capacity (LC) of 1.88 ± 0.01 µg piRNA/mg PEI‐PLA (Figure S6A,B). The PFH content was quantified by ^19^F‐NMR using Trifluoroacetic acid (TFA) as an internal standard to calculate the molar ratio between PFH and TFA. The apparent PFH retention efficiency was 56.52 ± 1.57% relative to the initially added PFH (Figure S6C,D), confirming the retention of a substantial amount of PFH within the nanoparticle system. Furthermore, the mass ratio of membrane proteins to polymeric cores was 16.4 ± 0.3 µg/mg PEI‐PLA, corresponding to 1.64 ± 0.03% (w/w) (Figure S6E). Furthermore, the FITC fluorescence quenching assay revealed a membrane coating efficiency of 73.27 ± 0.50%, confirming the effective coverage of nanoparticle surfaces by cardiomyocyte membranes (Figure S6F). All quantitative analyses were obtained from three independently prepared batches of nanoparticles.

**FIGURE 4:**
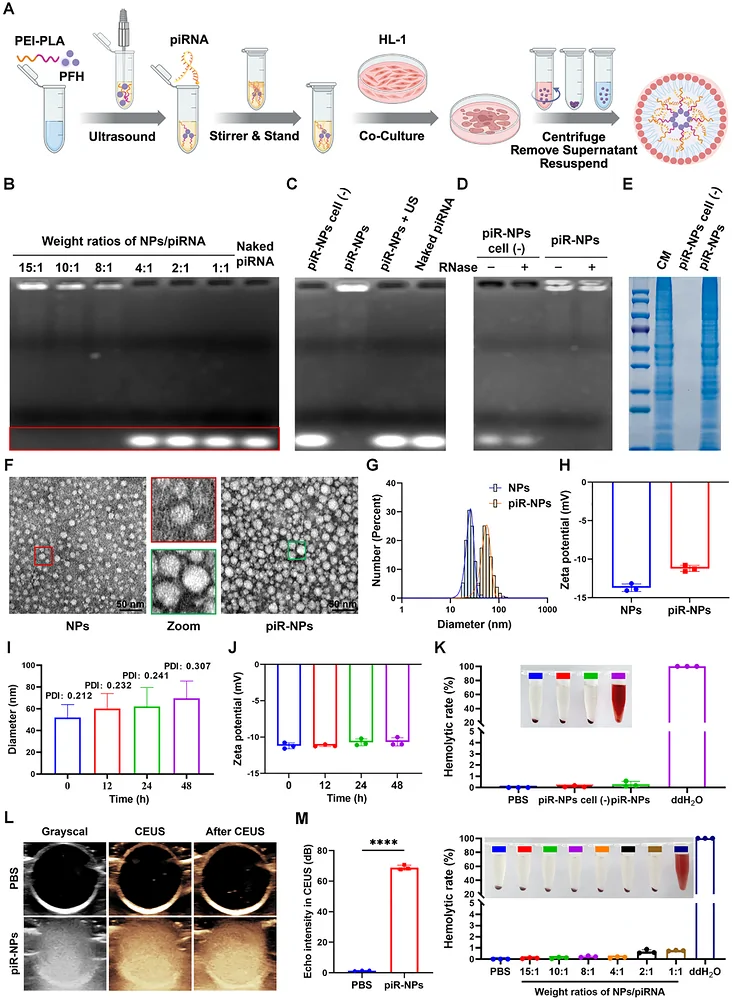
Preparation and characterization of NPs and piR‐NPs. (A) Schematic illustration of the preparation process of piR‐NPs. The schematic illustration was created using BioRender. (B) Agarose gel electrophoresis was used to detect binding at different NPs/piRNA mass ratios. (C) Agarose gel electrophoresis was used to detect nucleic acid loading and retention in piR‐NPs cell (−), piR‐NPs, piR‐NPs after ultrasound (piR‐NPs + US), and naked piRNA. (D) Allosteric nuclease treatment and nucleic acid degradation analysis of piR‐NPs cell (−) and piR‐NPs. (E) Coomassie blue staining was used to detect membrane components of piR‐NPs. (F) Representative TEM images of NPs and piR‐NPs. Scale bars: 50 nm. (G) DLS to measure particle size of NPs and piR‐NPs. (H) Zeta potential analysis of NPs and piR‐NPs (*n* = 3). (I) Particle size stability of piR‐NPs at different time points after preparation (*n* = 3). (J) Zeta potential stability analysis of piR‐NPs at different time points after preparation (*n* = 3). (K) Hemolysis assay to evaluate hemocompatibility of piR‐NPs and analysis of hemolysis rates of different NPs/piRNA formulations. PBS served as a negative control and ddH_2_O as a positive control (*n* = 3). (L, M) In vitro CEUS imaging of piR‐NPs and quantification of US echo intensity (*n* = 3). ^**^ *p* < 0.01, ^****^ *p* < 0.0001 compared with indicated groups.

The physicochemical properties of piR‐NPs were subsequently characterized. The morphology of the piR‐NPs, as observed through transmission electron microscopy (TEM), was uniformly spherical, well dispersed, and surrounded by an intact membrane layer with relatively even thickness (Figure 4F). These nanoparticles were approximately 56.10 nm in size, determined by dynamic light scattering (DLS) (Figure 4G). The zeta potentials of NPs and piR‐NPs were −13.71 ± 0.49 and −11.20 ± 0.40 mV, respectively, indicating a stable negatively charged surface in aqueous solution (Figure 4H). Stability testing over 48 h revealed no significant alterations in particle size, polydispersity index (PDI), or zeta potential (*p* > 0.05), with PDI values remaining below 0.4 throughout the observation period, underscoring the favorable physical stability of piR‐NPs (Figure 4I,J). Next, we further verified the orientation of cardiomyocyte membrane proteins on the nanoparticle surface. Proteinase K protection assay showed that the extracellular domain of the membrane marker protein N‐cadherin was reduced after Proteinase K treatment, whereas the intracellular domain was largely preserved in intact piR‐NPs. Following Triton X‐100‐mediated membrane disruption, the intracellular domain also became accessible to Proteinase K digestion and was markedly reduced, confirming the preserved membrane topology and integrity of cardiomyocyte membrane‐coated nanoparticles (Figure S6G,H). The hemolysis assay further confirmed the biocompatibility of piR‐NPs. Compared with the positive control group, no erythrocyte damage was observed in different preparation processes and at different concentrations of piR‐NPs, and the hemolysis rate was less than 1% (Figure 4K). piR‐NPs maintained good dispersion in PBS, saline, DMEM, and 15% FBS‐supplemented DMEM, and no cytotoxicity was observed when incubated with cells (Figure S5), further supporting their in vitro stability and cellular safety.

To evaluate the US responsiveness of piR‐NPs, in vitro US images were acquired in both grayscale and contrast‐enhanced ultrasound (CEUS) modes. Owing to the large acoustic impedance difference between PFH and water, piR‐NPs generated a relatively strong echogenic signal in grayscale mode. After US exposure, PFH underwent acoustic droplet vaporization (ADV), transitioning from the liquid to the gaseous phase and thereby increasing the local acoustic impedance difference [34]. This phase transition was observed as a homogeneous enhanced echogenic signal in CEUS mode (Figure 4L,M). Furthermore, the US responsiveness of piR‐NPs was systematically evaluated under different US intensities (0 W/cm^2^, 0.5 W/cm^2^, 1.0 W/cm^2^, 1.5 W/cm^2^, and 2.0 W/cm^2^). The results demonstrated that CEUS echo intensity progressively increased with increasing US intensity and reached a plateau at 1.5 W/cm^2^ (Figure S7A,B), suggesting that this intensity was sufficient to achieve near‐maximal acoustic activation of PFH‐containing nanoparticles. Subsequently, the dynamic monitoring capability of CEUS for piR‐NPs‐mediated therapy was further evaluated in the MI mouse model, aiming to explore its potential for monitoring ultrasound‐responsive nanoparticle behavior during treatment. Longitudinal CEUS imaging at different time points after LAD ligation enabled visualization of ultrasound signal changes during the therapeutic process. The CEUS signal changes showed a temporal association with LVEF recovery (Figure S7C,D). All of the above data support the successful construction of piR‐NPs that are stable, biocompatible, and US‐responsive.

### 2.5 PiR‐NPs Enhance Uptake, Inhibit Necroptosis, and Respond to US

To evaluate the cellular uptake efficiency of piR‐NPs, Cy5‐labeled piR‐NPs were incubated with cardiomyocytes pretreated with H_2_O_2_ for 8 h and subsequently subjected to US exposure. Immunofluorescence (IF) revealed that the red fluorescence signal in the cardiomyocyte membrane‐coated piR‐NPs was enhanced by two times as much as that in piR‐NPs cell (‐), and the fluorescence signal after US exposure was further enhanced by three times as much as that in piR‐NPs cell (‐), indicating that both the cardiomyocyte membrane coating and US could effectively promote the cellular uptake of nanoparticles (Figure 5A,B). Consistent with these observations, RT‐qPCR showed that piR‐NPs treatment increased piR‐1873 levels in cardiomyocytes, and US exposure further elevated its expression, demonstrating that functional piR‐1873 was successfully delivered (Figure 5C).

**FIGURE 5:**
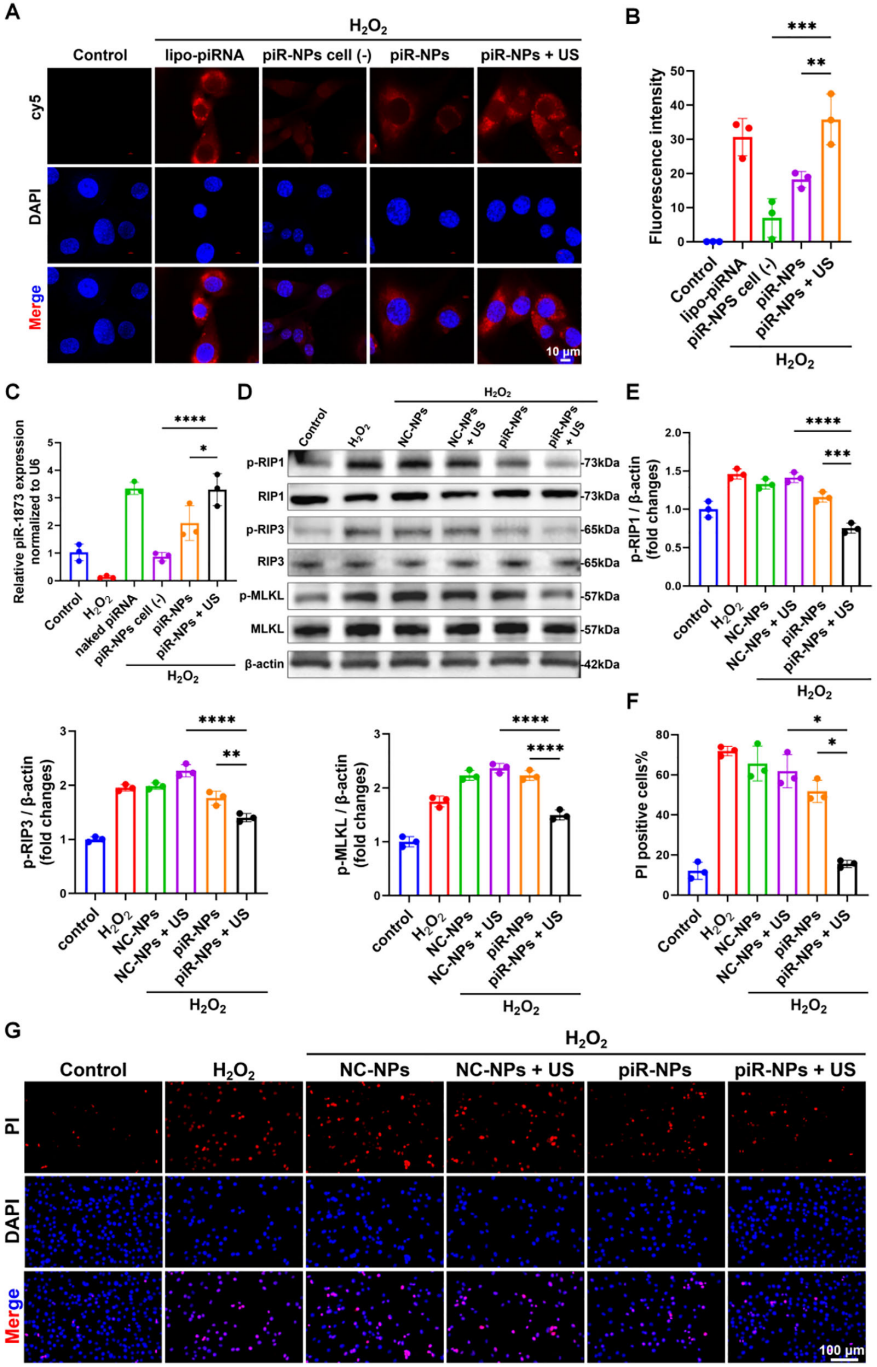
Cellular uptake of piR‐NPs protects cardiomyocytes. (A, B) Cardiomyocytes were pretreated with H_2_O_2_ for 8 h, then incubated with nanoparticles for 4 h, imaged by confocal microscopy, and quantified (*n* = 3). Scale bars: 10 µm. (C) RT‐qPCR was used to detect relative piR‐1873 expression in cardiomyocytes after H_2_O_2_ pretreatment and nanoparticle incubation (*n* = 3). (D, E) Western blotting was used to detect necroptosis‐related protein expression in cardiomyocytes after H_2_O_2_ pretreatment and nanoparticle incubation, and quantification (*n* = 3). (F, G) PI staining to assess cardiomyocyte necroptosis and quantification of the proportion of PI‐positive cells (*n* = 3). Scale bars: 100 µm. ^*^ *p* < 0.05, ^**^ *p* < 0.01, ^***^ *p* < 0.001, ^****^ *p* < 0.0001 compared with indicated groups.

The overall goal of this study was to establish an effective delivery system for piR‐1873 and to assess the therapeutic efficacy of nanoparticles in the laboratory setting. Negative control cardiomyocyte membrane‐coated nanoparticles (NC‐NPs) were constructed by replacing the mimic in piR‐NPs with a negative control (NC). Then, the protective effect of piR‐NPs on cardiomyocytes was verified by Western blot and PI staining. The results showed that H_2_O_2_ treatment increased the protein expression of p‐RIP1, p‐RIP3, and p‐MLKL and the proportion of PI‐positive cells (increased by 70% from 20%). piR‐NPs could markedly reverse these changes and reduce the levels of necroptosis‐associated proteins and the proportion of PI‐positive cells (decreased to 50%), and the effects were further enhanced by US exposure (the proportion of PI‐positive cells decreased to 20%) (Figures 5D‐G). These results suggest that piR‐NPs can be efficiently internalized by cardiomyocytes, successfully deliver functional piR‐1873, and that US exposure further enhances cellular accumulation, thereby effectively suppressing H_2_O_2_‐induced cardiomyocyte necroptosis.

### 2.6 PiR‐NPs Accumulate in Infarcted Myocardium with US‐Prolonged Retention

Next, we evaluated the in vivo targeting ability of piR‐NPs using a mouse MI model. The experimental procedure is shown in Figure 6A. The fluorescence intensity of Cy5‐labeled piR‐NPs in blood gradually decreased over time after intravenous injection. Notably, US treatment slowed the decline in fluorescence intensity and prolonged the detectable blood retention of piR‐NPs, with approximately 60% of the initial fluorescence signal remaining at 24 h after intravenous injection (Figure 6B,C). The peak enrichment of piR‐NPs in the cardiac region was observed 2 h after injection (Figure 6D,E); therefore, US exposure was applied to the cardiac region 2 h post‐injection in subsequent experiments. In vivo myocardial accumulation of piR‐NPs was monitored via left ventricular long‐axis echocardiography, with the echogenicity in the infarcted myocardium increasing to twice during US exposure than that before injection, and the echogenicity of the myocardial region was further doubled after US exposure (Figure 6F, G), indicating that US promotes piR‐NPs release and tissue penetration. To assess the targeting ability of piR‐NPs in the infarcted hearts, we compared the organ accumulation of MI mice [MI + piR‐NPs] with that of sham‐operated mice [Sham + piR‐NPs]. We found that fluorescence intensity in the heart region of MI mice was significantly higher than that in sham mice, and further enhanced by US exposure [MI + piR‐NPs + US] (Figure 6H,I). To explore the residence time of piR‐NPs in the cardiac region, in vivo imaging was performed in MI mice at 0 h, 4 h, 48 h, and 72 h after tail vein injection. US exposure significantly prolonged the retention time of piR‐NPs in the cardiac region, with noticeable fluorescence still detectable at 72 h post‐injection (Figure 6J,K). By dissecting the above three groups of mice, removing major organs (heart, liver, spleen, lung, and kidney), and performing *ex vivo* imaging analysis, we found that piR‐NPs were mainly distributed in the heart and liver, and US could further enhance cardiac accumulation (Figure 6L,M). Enhanced Cy5 fluorescence in the liver is a common observation in targeted delivery studies, probably because the liver is a major metabolic organ for lipids and proteins [35]. However, ex vivo fluorescence signals mainly reflect the distribution of nanoparticle‐associated signals and do not necessarily indicate efficient intracellular delivery or functional release of piR‐1873. Notably, ultrasound exposure was locally applied to the cardiac region in this study, and the enhanced nanoparticle uptake induced by ultrasound was therefore expected to occur predominantly in the myocardium. In addition, immunofluorescence staining of cardiac sections showed that the fluorescence intensity in the IZ was higher than in the RZ, and piR‐NPs colocalized with the cardiomyocyte marker cTNT (Figure 6N,O), suggesting enhanced localization of piR‐NPs within cTNT‐positive cardiomyocyte regions after US exposure. Collectively, these results demonstrate that piR‐NPs have good in vivo targeting and tissue selectivity, and cardiomyocyte membrane coating combined with US can significantly improve infarct‐specific targeting and retention of piR‐NPs in the infarcted myocardium.

**FIGURE 6:**
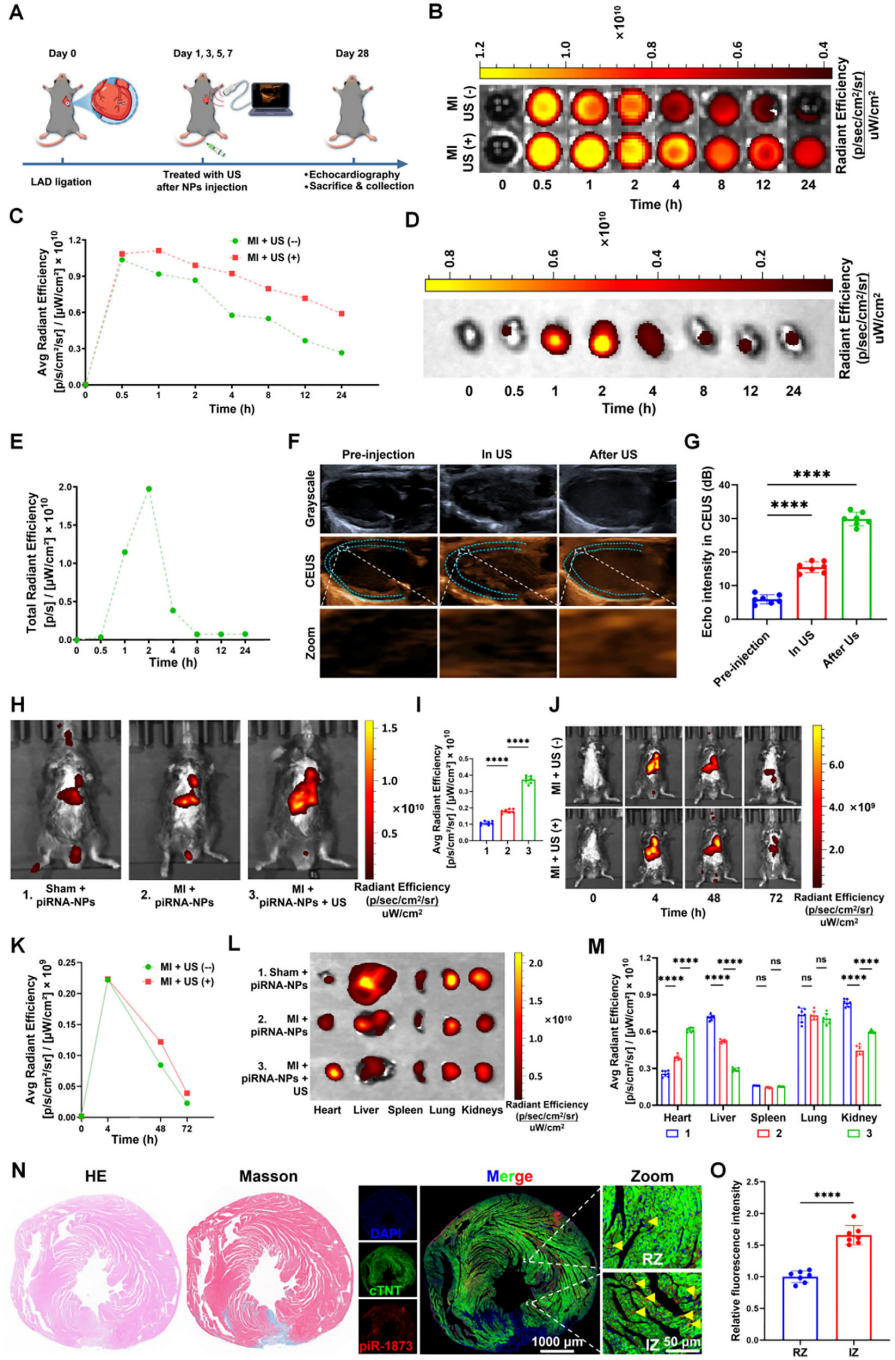
piR‐NPs were significantly enriched in the area of MI, and US could extend their enrichment time. (A) Schematic of in vivo delivery and imaging protocol in a mouse MI model. The schematic illustration was created using Adobe Illustrator. (B, C) Tail‐vein injection of piR‐NPs with or without US; blood collection at indicated time points; Cy5 fluorescence intensity measured and quantified. (D, E) Ex vivo optical imaging of Cy5 accumulation in the cardiac region of MI mice at different time points after tail vein injection and quantification analysis of fluorescence intensity (F, G) Ultrasound imaging of the cardiac region before and after piR‐NPs injection, including B‐mode, CEUS, and enlarged views, and quantification of US echo intensity (*n* = 7). (H, I) Live optical imaging of sham and MI model mice 2 h after piR‐NPs injection. Sham + piR‐NPs indicates tail vein injection of piR‐NPs in sham mice. MI + piR‐NPs represents tail vein injection of piR‐NPs in mice receiving MI without US. MI + piR‐NPs + US indicates that MI mice were irradiated with US after tail vein injection, and the fluorescence intensity was quantified (*n* = 7). (J, K) Live optical imaging at 0 h, 4 h, 48 h, and 72 h post‐injection with or without US, and quantification of fluorescence intensity in the precordial region (*n* = 7). (L, M) Ex vivo optical imaging of major organs (heart, liver, spleen, lung, and kidney) in sham and MI mice and quantification of mean fluorescence intensity (*n* = 7). (N) Fluorescence colocalization analysis of Cy5‐labeled piR‐1873 delivered by piR‐NPs with cardiomyocyte regions in infarcted myocardial tissue. (O) Quantitative analysis of fluorescence intensity between RZ and IZ (*n* = 7). Scale bars: 1000 µm; enlarged scale bar: 50 µm. ns: no significance, ^****^ *p* < 0.0001 compared with indicated groups.

### 2.7 PiR‐NPs Improve Cardiac Function and Structure, Further Enhanced by US

Before evaluating the therapeutic efficacy of piR‐NPs, we first examined whether US exposure or commercial transfection reagent‐mediated piR‐1873 delivery could influence cardiac function and tissue safety in MI mice. Echocardiographic assessment showed comparable LVEF, left ventricular fractional shortening (LVFS), left ventricular end‐systolic diameter (LVDs), and left ventricular end‐diastolic diameter (LVDd) values between the Sham and Sham + US groups, confirming the cardiac safety of the US parameters applied in this study. Moreover, MI + US mice did not exhibit significant improvement in cardiac function or cardiac structural remodeling at the endpoint (Figure S8A,B), suggesting that US exposure alone was insufficient to promote myocardial repair after MI. We next evaluated the therapeutic potential of piR‐1873 delivery using a commercial transfection reagent (MI + lipo‐piR). Administration of the MI + lipo‐piR formulation failed to improve LVEF, LVFS, or ventricular remodeling parameters. Histological analyses further showed that MI + lipo‐piR treatment did not reduce myocardial fibrosis or alleviate cardiomyocyte hypertrophy, as evidenced by HE/Masson staining and wheat germ agglutinin (WGA) staining, respectively (Figure S8A–F). Furthermore, elevated serum ALT and AST levels were observed in the MI + lipo‐piR group, accompanied by mild histological alterations in liver and kidney tissues, suggesting potential systemic safety concerns associated with the commercial transfection reagent‐mediated piR‐1873 delivery formulation (Figure S8G,H).

Based on these observations, we further investigated the in vivo targeting capability and therapeutic performance of piR‐NPs in the MI mouse model, with the dosing regimen illustrated in Figure 6A. Echocardiography was used to measure cardiac structure and function in each group at 28 days after infarction, and the effects of different treatments on cardiac function were then assessed. Cardiac function showed no obvious improvement in MI mice treated with NC‐NPs or NC‐NPs + US. In contrast, piR‐NPs treatment significantly increased LVEF and LVFS, while reducing LVDs and LVDd, indicating that piR‐NPs improved cardiac structure and systolic function after MI. Notably, the piR‐NPs + US group exhibited the most pronounced improvement in these parameters, suggesting that US further enhanced the in vivo therapeutic efficacy of piR‐NPs (Figure 7A,B). We then investigated the changes in heart samples harvested on day 28 after MI using TTC, H&E, Masson, and WGA staining. Compared with the NC‐NPs and NC‐NPs + US groups, piR‐NPs treatment reduced infarct size (Figure 7C,F), decreased myocardial fibrotic area (Figure 7D,G), and markedly reduced cardiomyocyte cross‐sectional area (Figure 7E,H), indicating structural recovery, improved cardiac function, and effective attenuation of ventricular remodeling. These protective effects were further improved in piR‐NPs + US treatment, confirming the beneficial role of piR‐NPs + US in alleviating post‐MI myocardial injury and remodeling. To test the ability of piR‐NPs + US to prevent cardiomyocyte necroptosis in MI mice, necroptosis‐related protein levels were assessed by Western blot. The protein levels of p‐RIP1, p‐RIP3, and p‐MLKL were decreased in the piR‐NPs group and were further reduced in the piR‐NPs + US group, indicating effective inhibition of cardiomyocyte necroptosis (Figure 7I,J). Collectively, combined treatment with piR‐NPs + US significantly attenuated ventricular remodeling and protected cardiac function after MI by delivering piR‐1873 and suppressing cardiomyocyte necroptosis.

**FIGURE 7:**
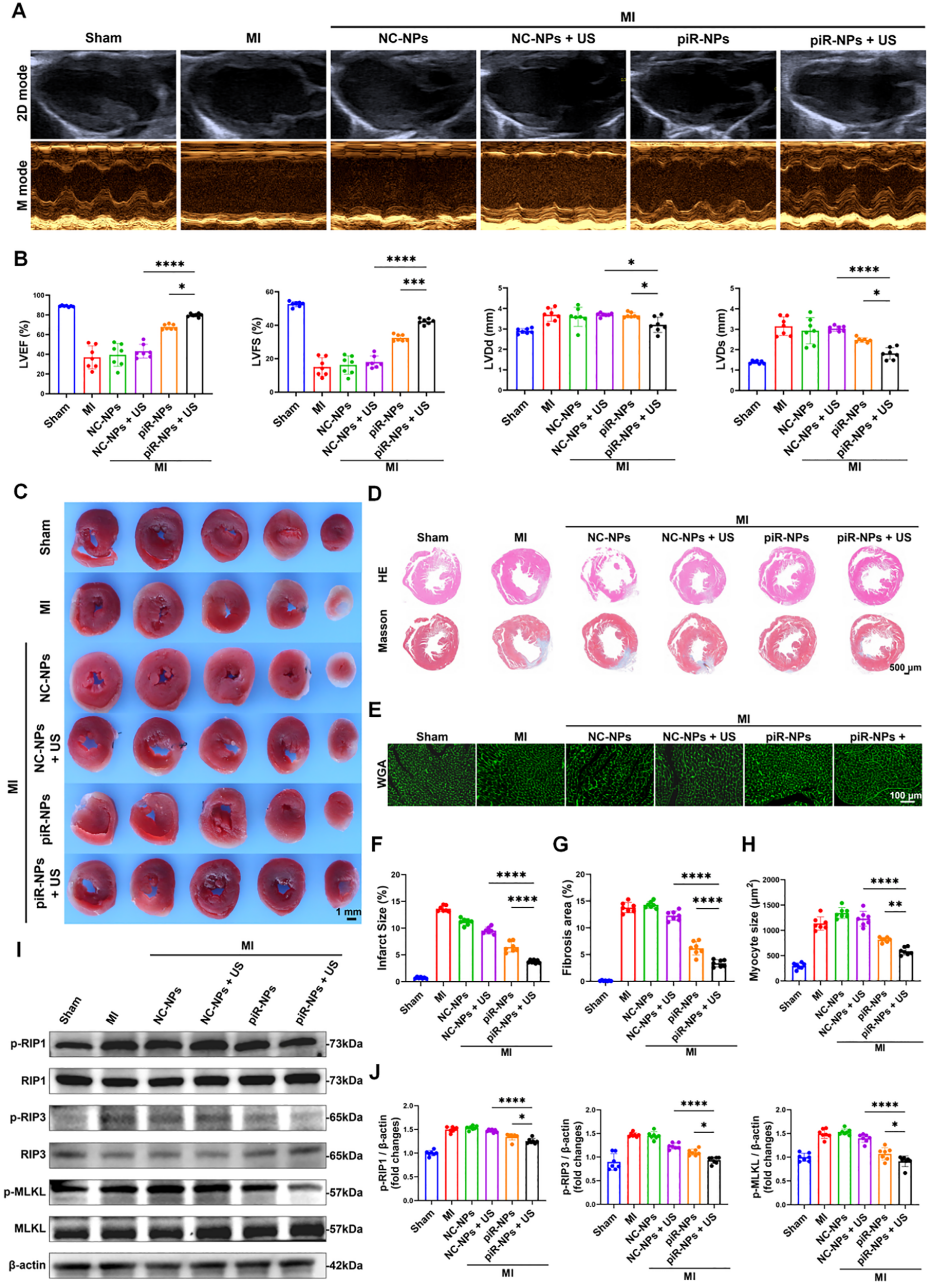
piR‐NPs preserve cardiac structure and function in MI mice. (A) Representative left ventricular long‐axis end‐diastolic 2D and M‐mode echocardiography from treatment and sham mice 28 days after MI. (B) Quantification analysis of left ventricular structural and functional parameters in each group: LVEF, LVFS, LVDd, and LVDs (*n* = 7). (C, F) TTC staining analysis of cardiac cross‐sectional sections of mice in each group, and quantitative analysis of myocardial infarct size (*n* = 3). Scale bars: 1 mm. (D, G) H&E and Masson staining images of cardiac tissue and quantification of fibrosis area (*n* = 7). Scale bars: 500 µm. (E, H) WGA staining images and quantification of cardiac myocyte area (*n* = 7). Scale bars: 100 µm. (I, J) Expression levels of necroptosis‐related proteins in myocardial tissue of treated and sham‐operated mice were detected by Western blot and quantified (*n* = 7). ^*^ *p* < 0.05, ^**^ *p* < 0.01, ^****^ *p* < 0.0001 compared with indicated groups.

### 2.8 PiR‐NPs Exhibit Excellent In Vivo Biosafety

Subsequently, to further evaluate the in vivo biosafety of piR‐NPs, H&E staining of the liver, spleen, lung, and kidney from MI mice treated with different piR‐NPs was conducted. The main organ structures of the mice in each group were intact without obvious pathological alterations (Figure 8A). In addition, liver function indicators, such as alanine aminotransferase (ALT) and aspartate aminotransferase (AST), renal function indicators, such as blood urea nitrogen (BUN) and creatinine (CREA), and lipid metabolism indicators, such as triglycerides (TG) and total cholesterol (CHO), were measured to be within the normal range in these groups (Figure 8B–G).

**FIGURE 8:**
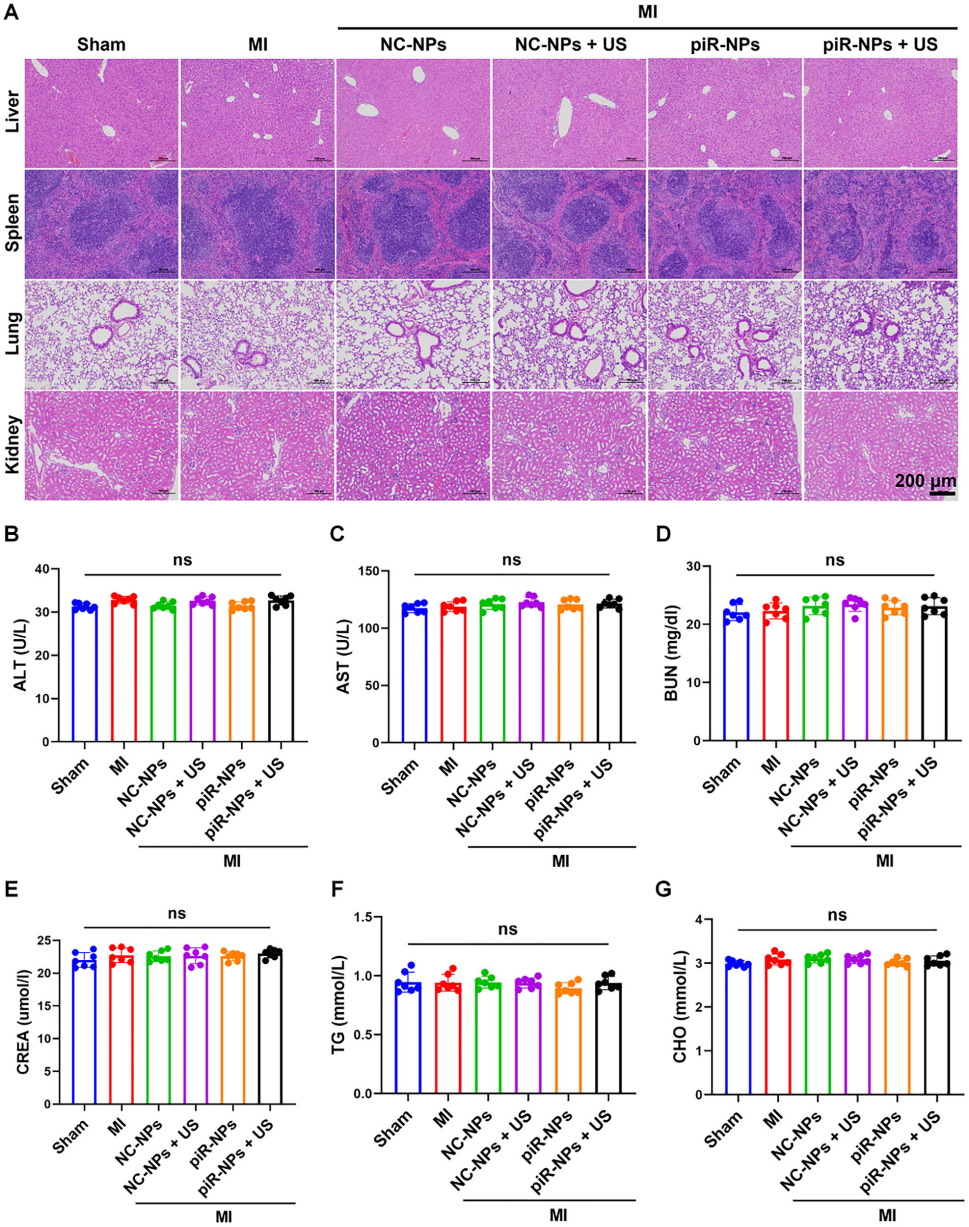
In vivo biosafety evaluation of piR‐NPs. (A) H&E staining images of major organs (liver, spleen, lung, kidney) of mice in each group (*n* = 7). Scale bars: 200 µm. (B‐G) Serum ALT (B), AST (C), BUN (D), CREA (E), TG (F), and CHO (G) levels (*n* = 7). ns: no significant difference among the indicated groups.

The above data proved that the piR‐NPs delivery system exhibits excellent in vivo biocompatibility and safety, with no observable hepatotoxicity or nephrotoxicity, supporting its potential for clinical translation.

## 3 Discussion

MI remains a major cause of morbidity and mortality worldwide, and accurate risk stratification and prevention of adverse ventricular remodeling after MI remain important clinical challenges. Traditional treatment methods are difficult to effectively prevent cardiomyocyte necrosis, reduce fibrosis, or improve cardiac function [36]. Therefore, the development of novel diagnostic biomarkers and in vivo delivery treatment strategies is essential to improve the survival rate of MI patients. In recent years, advances in nanotechnology and precision delivery strategies have provided new opportunities for cardiac repair. For example, mitochondrial‐mimetic therapies have been developed to restore mitochondrial homeostasis in dilated cardiomyopathy [37], nanotherapy‐engineered stem cell approaches have shown potential in remodeling the pathological microenvironment after myocardial injury [38], and engineered mitochondrial therapies have demonstrated efficacy in regulating inflammatory cell death pathways during myocarditis and fibrosis [39]. However, achieving precise delivery, spatiotemporally controlled release, and targeted regulation of specific pathological pathways remains a major challenge in cardiovascular therapy [40]. Small non‐coding RNA, particularly piRNA, is one of the most abundant types of small RNA in organisms. The 3′‐end methylation modification gives it a highly stable structure, which can resist nuclease degradation. Therefore, piRNA has natural advantages in molecular intervention and clinical transformation, but its specific role is still unclear [17]. In this study, we used large‐scale correlation analysis to reveal that serum piR‐1873 levels are significantly inversely correlated with MI biomarkers. Further ROC curve analysis demonstrated that piR‐1873 alone showed a moderate ability to discriminate MI (AUC = 0.8389), supporting its potential as a complementary molecular biomarker. Incorporation of piR‐1873 into the cTnI model increased the AUC from 0.9717 to 0.9767; although DeLong's test did not demonstrate a significant difference (*p* = 0.442), the combined model showed a numerical improvement, suggesting that piR‐1873 may provide additional molecular information beyond conventional myocardial injury markers rather than replace established diagnostic indicators. This limited improvement may be related to the high diagnostic performance of cTnI and the relatively small sample size. In the mouse MI model, piR‐1873 exhibited a distinct temporal pattern compared with cTnI and CK‐MB. piR‐1873 was persistently down‐regulated during the early stage of MI and reached the lowest level at 72 h, whereas cTnI and CK‐MB showed typical acute injury kinetics with early peaks followed by gradual declines. These findings suggest that piR‐1873 may reflect sustained molecular alterations during MI progression and serve as a complementary source of information to conventional cardiac injury markers. At the same time, because piR‐1873 quantification relies on qRT‐PCR, which requires longer processing time than rapid assays for cTnI or CK‐MB, its application in early clinical decision‐making may be limited. In addition, inflammation, comorbid cardiovascular conditions, and inter‐individual variability may influence piR‐1873 levels and contribute to potential false‐positive or false‐negative results. Further validation in larger cohorts, particularly including patients with non‐MI chest pain or other cardiovascular diseases, is warranted. Moreover, we found that piR‐1873 was significantly down‐regulated in the IZ region in patients with MI, animal models, and H_2_O_2_‐treated cardiomyocytes. The results showed that by targeted delivery of biomimetic phase‐changeable nanoparticles loaded with piR‐1873, cardiac function was significantly improved in MI mice. Myocardial cell necrosis and fibrosis were reduced. These findings establish piR‐1873 as a potential regulator of MI progression, with value as a complementary diagnostic biomarker and therapeutic target. It should be noted that blood samples were collected at admission before clinical intervention, whereas myocardial tissues represented the chronic remodeling stage after MI. Therefore, circulating and tissue piR‐1873 measurements reflect different temporal aspects of MI progression.

Bioinformatic predictions combined with experimental validation in this study indicate that Usp2 plays a critical role in the regulation of cardiomyocyte necroptosis. Interest in necroptosis has grown because of its importance in the evolution of the disease state and the response to therapy. Although 14 candidate genes were identified through bioinformatic screening, RNA pull‐down analysis showed stronger enrichment of Usp2, Hs3st3a1, and Atp2a3. However, transfection with the piR‐1873 inhibitor did not significantly alter Hs3st3a1 or Atp2a3 expression, and direct evidence linking these genes to cardiomyocyte necroptosis remains limited. Therefore, based on binding capacity, expression regulation, and functional validation, Usp2 was considered a functionally validated downstream effector mediating the protective effects of piR‐1873. In the irreversible injury of the cardiomyocytes in the infarcted area, necroptosis of the cells causes injury to the myocardium and limits the subsequent prognosis [41, 42]. Considering that necroptosis has been shown to be different from that of apoptosis, necroptosis involves the RIP1/RIP3/MLKL signaling axis, followed by breakdown of the membrane and the loss of intracellular constituents and the consequent amplification of inflammation and tissue damage [43]. As a deubiquitinating enzyme and ubiquitin‐specific protease, Usp2 may deubiquitinate and stabilize the necrosome core complex of RIP1, and thus be involved in the pathway RIP1/RIP3/MLKL by further phosphorylation and subsequent necrotic cell death [32]. Our data showed that piR‐1873 suppresses necroptosis through negative regulation of Usp2 expression. Rescue experiments showed that Usp2 overexpression partially reversed the protective effects of piR‐1873 on cardiomyocytes and restored the expression levels of necroptosis‐associated proteins, further supporting Usp2 as a downstream effector of piR‐1873. Mechanistically, piR‐1873 overexpression increased RIP1 ubiquitination and reduced the interaction between RIP1 and RIP3, whereas Usp2 overexpression reversed these effects. These findings suggest that piR‐1873 inhibits Usp2‐mediated RIP1 deubiquitination, thereby promoting RIP1 ubiquitination, limiting RIP1/RIP3 necrosome formation, and suppressing downstream RIP1/RIP3/MLKL signaling activation. This aligns with prior reports indicating that small RNA molecules can inhibit necroptosis by regulating key signaling axes, further supporting the cardioprotective role of piR‐1873. Unlike other piRNAs implicated in cardiac diseases, such as CHAPIR, which regulates cardiac hypertrophy via METTL3‐dependent m^6^A methylation [9], or HAAPIR, which promotes cardiomyocyte death via NAT10‐mediated ac^4^C acetylation [11], piR‐1873 exerts its cardioprotective effect by directly targeting Usp2 and suppressing necroptosis. This distinct mechanism highlights the functional diversity of piRNAs in the ischemic heart.

In order to better achieve in vivo delivery of piR‐1873 while addressing issues of degradation due to nucleases and low transmembrane delivery efficiency, our group designed and fabricated cardiomyocyte membrane‐coated piR‐NPs to encapsulate piR‐1873 in the core and PFH as a phase‐change material to facilitate US‐triggered release. piR‐NPs are significantly smaller in size (56.10 ± 11.81 nm) and are able to better circumvent the damaged endothelial barrier and penetrate the infarct area versus traditional microbubble destruction systems. This feature enhances accumulation and retention at the site, decreases loss prior to the target, increases the concentration of the drug intracellularly at the target, and ultimately increases the therapeutic effect. The cardiomyocyte membrane biomimetic strategy is a cornerstone of the innovations of this work. By utilizing natural cardiomyocyte membranes to coat the nanoparticles, the system is able to acquire the properties of the surface antigens from the source cells, thus facilitating active targeting while evading the immune system [44]. While the use of biomimetic membrane technology in the field of cardiovascular disease has been previously reported [45, 46], the use of cardiomyocyte membranes for piRNA‐targeted delivery in MI remains uncommon. Our experimental data demonstrated that, compared with uncoated piR‐NPs, cardiomyocyte membrane‐coated piR‐NPs exhibited significantly enhanced cellular uptake efficiency in H_2_O_2_‐treated cardiomyocytes, enabling precise drug delivery and validating the effectiveness of our cardiomyocyte membrane biomimetic strategy.

The incorporation of phase‐change material PFH, combined with US exposure, is another critical design element of this research. PFH remains liquid at room temperature, but upon US exposure, it undergoes a liquid‐to‐gas phase transition and generates gas bubbles that trigger controlled drug release [47]. The US parameters used in this study were optimized based on preliminary experiments and the acoustic requirements for PFH activation. While ensuring effective nanoparticle activation and drug release, this US exposure did not induce detectable cardiac tissue injury in either normal or MI mice, supporting its biosafety for in vivo application. The acoustic signals generated during PFH phase transition provide a noninvasive imaging readout of nanoparticle activation. This feature may facilitate the visualization of ultrasound‐responsive nanoparticle behavior and provide a potential approach for monitoring the therapeutic process. The cavitation effects generated during phase transition may induce reversible sonoporation of vascular endothelial cells and cardiomyocytes, thereby enhancing tissue penetration and intracellular delivery efficiency [48]. Our in vitro experiments confirmed that US significantly increased cellular uptake of piR‐NPs and intracellular piR‐1873 expression, while further strengthening the inhibitory effect on necroptosis. In vivo, US not only increased the initial accumulation of piR‐NPs within the infarcted myocardium, but also significantly prolonged their retention in the cardiac region. In addition, the prolonged detectable blood retention of piR‐NPs after US treatment may be associated with US‐mediated acoustic effects. US exposure may improve the dispersion of nanoparticles in the circulation and alter the dynamic distribution of piR‐NPs between the bloodstream and target tissues, thereby slowing the apparent decrease in circulating fluorescence signals. Consistent with these delivery findings, treatment with piR‐NPs combined with US improved cardiac function and structure in MI mice, reduced myocardial fibrosis area, and significantly decreased the expression of necroptosis‐related proteins. Notably, US exposure alone failed to improve cardiac function or remodeling after MI, while commercial transfection reagent‐mediated piR‐1873 delivery showed limited therapeutic benefits and potential systemic safety concerns. These findings suggest that the therapeutic efficacy mainly arises from the synergistic combination of piR‐NPs‐mediated delivery and US‐triggered activation rather than US exposure or commercial transfection reagent‐mediated piR‐1873 alone. The accumulation of piR‐NPs in the liver is consistent with the biodistribution characteristics of many intravenously administered nanomaterials, which may be attributed to the role of the liver in nanoparticle metabolism and clearance. However, fluorescence‐based biodistribution primarily reflects the accumulation of Cy5‐associated nanoparticle signals within organs and does not directly demonstrate intracellular release of piR‐1873 in hepatocytes or subsequent regulation of hepatic Usp2. In this study, US exposure was locally applied to the cardiac region, which may enhance nanoparticle uptake and piR‐1873 delivery in the infarcted myocardium. Nevertheless, the functional delivery and potential biological effects of piR‐NPs in non‐target organs after systemic administration require further investigation. Given that Usp2 is broadly expressed and involved in multiple biological processes, including cell‐cycle regulation, p53 signaling, and metabolic regulation, unintended modulation of Usp2 in non‐cardiac tissues cannot be completely excluded after systemic piR‐1873 delivery. Further studies investigating tissue‐specific piR‐1873/Usp2 regulation will be important to better define the systemic pharmacological profile of this platform. In addition, the MI model in this study was established in 6‐ 8‐week‐old mice, whereas clinical MI predominantly occurs in older populations. Age‐related differences in inflammatory responses, vascular permeability, and cardiac repair capacity may influence nanoparticle delivery efficiency and therapeutic responses. Therefore, further validation in aged animal models is needed to evaluate the translational potential of piR‐NPs. Such a spatiotemporally controllable release pattern is difficult to achieve with conventional administration strategies and represents a central advantage of the present system. By using external US to trigger targeted disruption of piR‐NPs within the infarct region and leveraging the unique acoustic cavitation effect, the local concentration of therapeutic piRNA can be maximized. This delivery strategy may therefore also be adaptable to other ischemic heart diseases.

Overall, we developed and constructed a piRNA nanodelivery system that integrates a biomimetic membrane strategy with US‐responsive nucleic acid delivery. Cardiomyocyte membrane‐based biomimetic nanoparticles not only ensured efficient delivery of piR‐1873, but also enabled spatiotemporally controllable release under US exposure, thereby significantly improving cardiac structure and function in MI mice while maintaining favorable in vivo safety. The research provides an advanced understanding of the role of piRNAs in MI therapy and details the translational basis for using RNA‐based precision therapeutics in cardiovascular disorders.

Although advancements have been made, a number of limitations should be considered. First, further validation in larger cohorts, particularly including patients with non‐MI chest pain or other cardiovascular diseases, is warranted to determine the clinical utility of piR‐1873 as a complementary biomarker for MI. Second, although piR‐1873 expression was lower in NCFs than in NVCMs, it was not completely absent. However, the present study focused exclusively on its regulatory role in cardiomyocytes, and its function in cardiac fibroblasts remains to be further investigated. In this study, a single‐dose regimen of piR‐NPs was used for in vivo therapeutic evaluation, which was selected based on effective dosage ranges reported in previous nucleic acid delivery strategies. Nevertheless, comprehensive dose‐response studies are warranted to further define the optimal therapeutic window, long‐term safety profile, and translational potential of piR‐NPs. Moreover, the accumulation of piR‐NPs in non‐target organs such as the liver suggests potential extra‐cardiac exposure. Although no obvious systemic toxicity was observed under the current treatment conditions, tissue‐specific molecular effects caused by systemic piR‐1873 delivery cannot be completely excluded and require further investigation. In addition, although additional characterization confirmed substantial membrane coverage and preserved membrane protein topology of piR‐NPs, the current preparation strategy cannot completely exclude the presence of a minor fraction of incompletely coated or uncoated nanoparticles. Finally, left ventricular function was assessed by M‐mode echocardiography in mice. Due to the small size and rapid heart rate of mice, stable acquisition of the apical two‐ and four‐chamber views required for the clinical Simpson method remains challenging, which may introduce measurement variability. Future studies using advanced small‐animal 3D echocardiography may further improve the accuracy of cardiac functional assessment.

## 4 Conclusion

This study is the first to systematically reveal that piR‐1873 is significantly down‐regulated in MI patients, mouse models, and H_2_O_2_‐treated cardiomyocytes, and that it exerts cardioprotective effects by regulating cardiomyocyte necroptosis through the Usp2‐mediated RIP1/RIP3/MLKL signaling axis. Moreover, serum piR‐1873 levels exhibited diagnostic value for MI. On this basis, we successfully constructed cardiomyocyte membrane‐coated piR‐NPs that, upon US exposure, enabled efficient targeted delivery and spatiotemporally controlled release of piR‐1873, thereby suppressing cardiomyocyte necroptosis in vitro and in vivo, improving cardiac structure and function, and exhibiting favorable biosafety. These findings not only provide a new perspective for the application of small ncRNAs in cardiovascular disease, but also offer a representative framework for the design of precision targeted delivery systems with considerable clinical translational potential.

## 5 Experimental Section

### 5.1 Clinical Sample Collection

Clinical myocardial tissue and blood samples were collected at the Affiliated Hospital of Qingdao University between March 2024 and December 2025. The study protocol was approved by the Ethics Committee of Qingdao University Affiliated Hospital (Approval No. QYFY WZLL 50058). Written informed consent was obtained from all enrolled individuals. Myocardial tissue samples were collected from patients with post‐MI ventricular aneurysm who underwent heart transplantation. During surgery, paired myocardial specimens were obtained from the IZ, characterized by fibrotic and necrotic alterations, and the RZ, representing relatively preserved non‐infarcted myocardium, for subsequent histological analysis and piR‐1873 expression evaluation. Venous peripheral blood was collected from two groups: patients diagnosed with MI (MI group) and healthy controls free from any prior cardiovascular disease (Normal group). In the MI group, blood samples were obtained at the time of hospital admission before any clinical intervention. Blood samples were collected to detect the expression level of piR‐1873 and clinical indicators related to myocardial injury, including cTnI, NT‐proBNP, and CRP. Resected myocardial specimens were immediately snap‑frozen in liquid nitrogen. For serum isolation, peripheral blood samples were placed into serum separation tubes and centrifuged at 3,000 rpm for 10 min at 4°C. All tissue and serum samples were subsequently stored at −80°C pending piRNA extraction and further experimental analyses.

### 5.2 Cell Culture and Transfection

Mouse HL‐1 cardiomyocytes (purchased from Shanghai Biowing Applied Biotechnology Co., Ltd., China) were grown in DMEM (Bios, Beijing) with 10% fetal bovine serum (Procell, Wuhan), 1% penicillin‑streptomycin (Meilunbio, Dalian), at 37°C in a humidified 5% CO_2_ incubator. For transfection, cells were seeded into plates and allowed to reach 60–80% confluence. Lipofectamine 3000 (Invitrogen, Carlsbad, CA, USA) was used to deliver piRNA mimic or inhibitor into the cells, following the supplier's protocol. Sequences for the mimic and inhibitor are listed in Table S3.

### 5.3 RT‐qPCR

Total RNA from tissues and cells was extracted using RNA‑easy Isolation Reagent (Vazyme, Nanjing, China). Tissue samples were homogenized with steel beads in a grinder. For piRNA, cDNA was synthesized with the miRNA First‑Strand cDNA Synthesis Kit (Accurate Biology, Changsha, China); for mRNA, with HiScript III First‑Strand cDNA Synthesis Kit (+gDNA wiper, Vazyme). RT‑qPCR was run on an AriaMx System (Agilent) using SYBR Green Master Mix (Yeasen). Relative expression (2^ΔΔCt^) was calculated using U6 (piRNA) or GAPDH (mRNA) as controls. Primer sequences are provided in Table S4. The stability of U6 and GAPDH as reference transcripts was validated by comparing their raw Ct values among different experimental groups (Figure S9).

### 5.4 Western Blot Analysis

Total protein was extracted with RIPA buffer (Solarbio) containing PMSF and protease inhibitor cocktail (ABmole). Protein concentration was measured by the bicinchoninic acid (BCA) kit (EpiZyme). Equal protein loads were separated by 12.5% SDS‑PAGE (EpiZyme) and transferred to PVDF membranes (Millipore). After blocking (5% BSA/TBST, 1 h, RT), membranes were incubated with primary antibodies (overnight, 4°C), then HRP‑secondary antibodies (1 h, RT). Bands were visualized with Fusion Solo S (Vilber) and quantified by ImageJ (NIH). For western blot quantification, target proteins and β‐actin were analyzed using protein lysates prepared from the same experimental samples. One target protein and β‐actin were detected on the same membrane because their molecular weights were sufficiently separated, and the corresponding bands were analyzed from different regions of the membrane. The remaining target proteins were detected using independently prepared gels and membranes, with β‐actin loading controls obtained from matched samples. No sequential stripping and re‐probing procedures were performed. Band intensities were quantified by densitometric analysis using ImageJ software (NIH), and the relative expression levels of target proteins were normalized to the corresponding β‐actin loading controls from matched samples. Antibody details are listed in Table S5.

### 5.5 FISH and IF

FISH was performed using a kit from Beyotime Biotechnology (Shanghai, China). Samples were fixed in 4% paraformaldehyde (PFA; Solarbio, Beijing, China) for 20 min, permeabilized with proteinase K (10 µg/mL) for 10 min, and subjected to pre‐hybridization at 37°C for 30 min, followed by overnight hybridization at 37°C. After washing with graded SSC buffers, nuclei were counterstained with DAPI (Solarbio, Beijing, China). For combined FISH and IF staining, tissue sections were blocked with 3% BSA and incubated with anti‐cTnT primary antibody (1:200), followed by Fluorescein Isothiocyanate (FITC)‐conjugated secondary antibody. Images were obtained after mounting with an anti‐fade medium containing DAPI. Fluorescence images were acquired using a Nikon upright fluorescence microscope (Nikon, Tokyo, Japan). Probe sequences are listed in Table S6.

### 5.6 PI Staining

Cells cultured on coverslips were washed with PBS, stained with PI (1:1000) at 37°C for 20 min in the dark, then fixed with 4% PFA for 20 min, and counterstained with DAPI (1 µg/mL) for 5 min. Fluorescence images were obtained using a fluorescence microscope.

### 5.7 Cell Proliferation Assays (CCK‐8 and EdU)

Cell viability was assessed with a CCK‑8 kit (Meilunbio, Dalian, China). Cells in 96‑well plates received the indicated treatments for 24 h, then 10 µL of CCK‑8 reagent was added to each well. After 2 h of incubation at 37°C, absorbance at 450 nm was measured. DNA synthesis was evaluated using an EdU‑647 kit (CX004; EpiZyme, Shanghai, China). Growing cells were exposed to 10 µm EdU for 2 h (37°C), fixed with 4% PFA (15 min), permeabilized with 0.3% Triton X‑100 (5 min), and subjected to a Click reaction (30 min, dark). Nuclei were stained with DAPI. EdU‑positive cell percentages were calculated via ImageJ.

### 5.8 TUNEL Assay

Apoptosis was evaluated with a One Step TUNEL kit (C1089; Beyotime, Shanghai). Cells were fixed in 4% PFA (20 min), permeabilized with 0.2% Triton X‐100 (15 min), then incubated with TUNEL mixture for 60 min at 37°C in darkness. DAPI stained the nuclei. ImageJ software quantified the apoptotic rate.

### 5.9 RNA Sequencing

HL‐1 cells received transfection of piRNA‐1873 mimics or NC mimics. Total RNA was extracted, and sequencing libraries were prepared using the NEBNext Ultra RNA Library Prep Kit (NEB, Ipswich, MA, USA) per the manufacturer's protocol. Cluster generation employed the TruSeq PE Cluster Kit v3‐cBot‐HS (Illumina, San Diego, CA, USA). Paired‐end sequencing ran on an Illumina NovaSeq 6000 system (Illumina) with an S4 flow cell. The raw and processed RNA‐sequencing data generated in this study have been deposited in the NCBI Gene Expression Omnibus (GEO) database under accession number GSE343719.

### 5.10 Dual‐Luciferase Reporter Assay

To verify piRNA‑1873 binding to Usp2, WT or Mut 3′‑UTR segments of Usp2 harboring the predicted piRNA‑1873 recognition sites were inserted into the pmirGLO vector (Genechem, Shanghai, China) via Sall and Sacl. HL‑1 cells received co‑transfection of these luciferase constructs together with piRNA‑1873 mimics or NC mimics using Lipofectamine 3000 (Invitrogen, Carlsbad, CA, USA). After 48 h, luciferase signals were measured with the Firefly & Renilla‑Glo Luciferase Assay Kit (Genechem) following the provided protocol. The ratio of firefly to Renilla luminescence represented relative luciferase activity.

### 5.11 Biotin‐Labeled RNA Pull‐Down Assay

HL‑1 cells (2 × 10^7^) were lysed in buffer (20 mm Tris‑HCl, pH 7.5, 200 mm NaCl, 2.5 mm MgCl_2_, 1% Triton X‑100, 1 mm DTT, 60 U/mL RNase inhibitor, PMSF; all from Solarbio except RNase inhibitor from SparkJade). Streptavidin beads, pre‑blocked with yeast tRNA/BSA, were incubated with 20 µm biotinylated probes (4°C, 3 h). Lysates were added to bead‑probe complexes22 and rotated overnight (4°C). After five high‑salt washes, bound RNA was extracted with TRIzol and subjected to RT‑qPCR.

### 5.12 Preparation and Characterization of piR‐NPs

The piR‐NPs were prepared by mixing 150 µL PFH, 600 µL PEI‐PLA, and 2250 µL PBS (total volume: 3 mL) in a centrifuge tube. The concentration of the PEI‐PLA solution was 50 mg/mL, resulting in 30 mg of PEI‐PLA in 600 µL of solution. The density of PFH was 1.669 g/mL, and 150 µL of PFH corresponded to a mass of 250.35 mg. The mixture was emulsified in an ice bath using a probe sonicator at 70% amplitude for 5 min (5 s on, 5 s off). The emulsion was left to stabilize at 4°C overnight. The supernatant was obtained and then passed through a 0.45 µm membrane to separate large aggregates. Each 1 mL of nanoparticle suspension corresponded to 10 mg PEI‐PLA and 50 µL PFH. To prepare piRNA‐loaded nanoparticle cores, the filtered nanoparticle suspension (1 mL, containing 10 mg PEI‐PLA and 50 µL PFH) was incubated with 20 µg Cy5‐labeled piRNA mimics (consistent with concentrations commonly applied in small RNA mimic‐mediated functional studies) at room temperature for 20 min to form piR‐NPs complexes through electrostatic interactions. Before membrane coating, unbound free piRNA was separated from the piR‐NPs complexes using ultrafiltration centrifugation. The piR‐NPs complexes were subsequently co‐incubated with HL‐1 cells for 8 h to allow cellular uptake and potential membrane coating or modification. Each batch of preparation used [2.42 ± 0.60] × 10^6^ HL‐1 cells with an approximately 80% cell density. After the formation of the complexes, membrane coating and uptake of the nanoparticles by the cells were determined after 8 h. The resulting culture supernatant from the 8‐h cell culture was collected. This was then subjected to 30 min centrifugation at 12,000 × g and 4°C. The resultant supernatant was discarded, and the pellet was obtained, containing the nanoparticles. This was then washed with 100 µL PBS and finally filtered through a 0.22 µm membrane.

Morphology was analyzed by negative staining with phosphotungstic acid along with imaging by a TEM (JEM‐2100F, JEOL, Tokyo, Japan). To assess the particle size, PDI, or zeta potential, a BI‐90Plus particle size analyzer (Brookhaven Instruments, Holtsville, NY, USA) was utilized. Hemocompatibility was assessed by incubating piR‐NPs at varying NP/piRNA weight ratios with mouse red blood cells (RBCs) for 1 h at 37°C, followed by centrifugation at 3000 rpm for 15 min. The hemolysis rate (%) was calculated using the following The hemolysis rate was calculated as follows: Hemolysis (%) = (OD_sample_—OD_PBS_) / (OD_ddH2O_—OD_PBS_) × 100%, with PBS and double‐distilled water (ddH_2_O) as negative and positive controls, respectively.

### 5.13 EE and LC of piRNA

Cy5‐labeled piRNA was mixed with the nanoparticle suspension, and the mixture was transferred into a 10 kDa ultrafiltration centrifuge tube. After centrifugation, unbound free piRNA in the filtrate was collected and quantified using a Cy5‐piRNA fluorescence standard curve. Before fluorescence measurement, samples were diluted 10‐fold to ensure that the fluorescence intensity fell within the linear range of the Cy5‐piRNA calibration curve. The Cy5‐piRNA standard curve was generated using a series of Cy5‐piRNA concentrations (0 µg/mL, 0.025 µg/mL, 0.05 µg/mL, 0.1 µg/mL, 0.2 µg/mL, and 0.4 µg/mL). Fluorescence intensity was measured using a fluorescence microplate reader in black 96‐well plates. The loading capacity was expressed as the amount of piRNA loaded per milligram of PEI‐PLA. The encapsulation efficiency and loading capacity of piRNA were calculated based on the initial amount of added piRNA and the amount of unbound free piRNA according to the following equations:

$$piRNA\;\;EE\left(\%\right)=\left(M_{total\;\;piRNA}-M_{free\;\;piRNA}\right)/M_{total\;\;piRNA}\times 100\%$$

$$piRNA\;\;LO=\left(M_{total\;\;piRNA}-M_{free\;\;piRNA}\right)/M_{PEI-PLA}$$

### 5.14 Quantification of PFH content and PFH retention efficiency by ^19^F‐NMR

Quantitative ^19^F‐NMR was used to determine the PFH content in piR‐NPs. TFA (10 µL, 99.9%) was used as an internal standard. The amount of TFA was calculated based on its density (1.489 g/mL) and molecular weight (114.02 g/mol), corresponding to 130.461 µmol. The molar ratio of PFH to TFA was calculated based on the integrated ^19^F‐NMR signals using the following equation: n(PFH) / n(TFA) = [I(PFH)/6] / [I(TFA)/3]. Based on the integration results, the PFH content in 10 µL of piR‐NPs suspension was calculated as 4.719 mg. After volume normalization, the total PFH amount in the final piR‐NPs suspension was estimated to be 47.188 mg. Using the initially added PFH amount (50 µL, 83.45 mg) as the reference, the apparent PFH retention efficiency was calculated according to the following equation:

$$\begin{array}{cc} & PFH\;retention\;efficiency\;\left(\%\right)\\ & =PFH\;content\;in\;final\;nanoparticle\;suspension/initial\;PFH\\ & \times 100\% \end{array}$$

The detailed quantitative analysis of PFH content and apparent retention efficiency is summarized in Table S7.

### 5.15 Quantification of Membrane‐Associated Proteins and Analysis of Membrane Protein‐to‐Polymer Core Mass Ratio

Equal amounts of final membrane‐coated piR‐NPs were lysed using a buffer containing 1% Triton X‐100 to disrupt the lipid membrane structure and release membrane‐associated proteins. The total protein content associated with nanoparticles was quantified using the BCA assay. Meanwhile, uncoated PEI‐PLA/PFH nanoparticle cores, termed piR‐NPs (cell‐), were used as background controls to correct for potential interference from nanoparticle materials during protein quantification. The amount of membrane‐derived proteins was calculated by subtracting the background protein content detected from uncoated nanoparticle cores from the total protein content measured in membrane‐coated nanoparticles. The membrane protein‐to‐polymer core mass ratio was calculated using the following equation:

$$\begin{array}{cc} & Membrane\;\;protein-to-core\;\;ratio\\ & =\left(M_{piR-NPs}-M_{piR-NPs(cell-)}\right)/M_{PEI-PLA} \end{array}$$

### 5.16 Evaluation of Membrane Coating Efficiency by FITC Fluorescence Quenching Assay

To quantitatively evaluate the coating efficiency of cardiomyocyte membranes on nanoparticle cores, a FITC fluorescence quenching assay was performed. FITC was first dissolved in DMSO at a concentration of 1 mg/mL and stored protected from light. FITC was then conjugated onto the surface of PEI‐PLA nanoparticle cores and incubated overnight at room temperature in the dark. Subsequently, cardiomyocyte membrane‐coated nanoparticles (FITC‐membrane‐coated NPs) were prepared according to the above‐described procedure. FITC‐labeled uncoated nanoparticle cores (FITC‐core) were used as controls. Equal amounts of FITC‐labeled nanoparticles were incubated with anti‐FITC antibodies for 15 min in the dark, and changes in fluorescence intensity were measured. A FITC fluorescence standard curve was established using FITC solutions with different concentrations, and fluorescence intensity was converted into FITC concentration based on the calibration curve. The membrane coating efficiency was calculated according to the reduction in exposed FITC after membrane coating using the following equation:

$$\begin{array}{cc} & Membrane\;\;coating\;\;efficiency\left(\%\right)\\ & =\left(C_{piR-NPscell\left(-\right)FITC}-C_{piR-NPs\;\;FITC}\right)/C_{piR-NPscell\left(-\right)\;\;FITC} \end{array}$$

where C_piR‐NPscell(‐) FITC_ represents the amount of exposed FITC on uncoated nanoparticle cores, and C_piR‐NPs FITC_ represents the amount of exposed FITC after membrane coating.

### 5.17 Proteinase K Protection Assay

To verify the topology and orientation of membrane proteins after cardiomyocyte membrane coating, a Proteinase K protection assay was performed. Membrane‐coated piR‐NPs were treated with Proteinase K alone or pretreated with Triton X‐100 followed by Proteinase K digestion. Proteinase K was used to degrade membrane protein domains exposed on the nanoparticle surface, whereas Triton X‐100 was applied to disrupt the lipid bilayer and expose all membrane proteins to enzymatic digestion. After termination of the reaction, Western blotting was performed to detect changes in the extracellular and intracellular domains of N‐cadherin, thereby evaluating membrane protein orientation and membrane coating integrity.

### 5.18 Coomassie Brilliant Blue Staining and Gel Retardation Analyses

Protein extraction from piR‐NPs was done using RIPA buffer (Solarbio, Beijing, China), followed by protein separation using SDS‐PAGE. Coomassie Brilliant Blue (Solarbio) was used to stain protein bands for visualization. To study piRNA loading, membrane‐coated piR‐NPs and uncoated nanoparticles [piR‐NPs cell(‐)]were made at various NP/piRNA weight ratios, incubated at 37°C for 20 min, and subsequently loaded into agarose gels containing EB. Electrophoresis was conducted using TAE buffer (Solarbio) at 100 V for 25 min with a Mini‐Sub Cell GT system (Bio‐Rad, USA), then piRNA bands were captured using a GDS‐8000 imaging system.

### 5.19 Cellular Uptake Assay

HL‑1 cells were seeded onto glass coverslips. For pathological stimulation, cells were exposed to H_2_O_2_. Then, cells received either Cy5‑labeled piRNA‑NPs or piRNA‑NPs cell(‑) for 4 h at 37°C. Cy5‑piRNA delivered by Lipofectamine 3000 served as a positive control. After incubation, cells were washed with PBS, fixed with 4% PFA (20 min), and mounted in DAPI‑containing antifade medium. Fluorescence images were captured using a confocal laser scanning microscope (Leica, Germany), and mean fluorescence intensity was quantified with ImageJ.

### 5.20 Animal Model

All procedures involving animals were approved by the Animal Experiment Ethics and Guidelines Committee of Qingdao University (No. 20240701C576J4820240731089) and complied with China's Animal Protection Law as well as the Laboratory Animal Care and Use Guidelines (National Health Commission). Male C57BL/6J mice (6‐8 weeks old) were obtained from Beijing HFK Bioscience Co., Ltd. (Beijing, China) and housed at Qingdao University's Animal Center. MI was induced by permanent ligation of the LAD coronary artery under sodium pentobarbital anesthesia (50 mg/kg) with mechanical ventilation. Successful ligation was confirmed by visible blanching of the myocardium. Successful ligation was defined by myocardial blanching. Sham‐operated mice underwent identical procedures without ligation. MI mice were randomly assigned to treatment groups and received intravenous tail injections on days 1, 3, 5, and 7 post‐MI. For in vivo administration, mice received tail vein injection of piR‐NPs containing 2.8 µg of piR‐1873 per mouse (approximately 112.8 µg/kg based on a mean body weight of 25 g). The injection volume was controlled at 100 µL to 200 µL per mouse. The dose chosen was determined based on previously reported effective doses for nucleic acid delivery systems. The drug was administered slowly through the tail vein. A US study was conducted to confirm the treatment in the precordial region using the VINNO 10 system (VINNO Technology, Suzhou, China) to trigger the release of the medication, at a frequency of 1.0 MHz, power density of 1.5 W/cm^2^, for a duration of 1 min, 2 h following each injection. At day 28 after LAD ligation, cardiac function was assessed using a high‐frequency US system (VINNO LAB, VINNO Technology, Suzhou, China) equipped with an X10‐23L linear‐array transducer (23 MHz). Mice were anesthetized with 1–2% isoflurane, and B‐mode and M‐mode images were acquired from the parasternal long‐axis view. LVEF, LVFS, LVDd, and LVDs were measured from M‐mode images, with values averaged from three consecutive cardiac cycles. Following MI and on Day 28, blood was collected to study the toxicity of major organs (heart, liver, spleen, lung, and kidney).

### 5.21 In Vivo Biodistribution and Pharmacokinetics Analysis

MI mice (6‐8 weeks) were injected via the tail vein with Cy5‐labeled piR‐NPs (0.625 mg/kg) on day 3 post‐MI. Mice were sacrificed for independent subgroup analysis at 0 h, 0.5 h, 1 h, 2 h, 4 h, 8 h, 12 h, and 24 h post‐administration. An IVIS imaging system (Caliper Life Sciences, Hopkinton, MA, USA) captured the fluorescence images. Around 50 µL of blood was drawn at the stipulated time intervals and placed in black 96‐well plates, where the fluorescence intensity was recorded. Major organs were collected from the study for ex vivo imaging. Nanoparticle accumulation was quantified for the collected hearts. ImageJ software was used to evaluate all of the data.

### 5.22 Histological and Immunofluorescence

Fresh hearts were frozen at −20°C for 30 min, and then sliced into 2 mm sections. The sections were incubated in 1% TTC at 37°C in the dark for 15 min. After the incubation, stained red areas indicated viable myocardium, and pale areas indicated infarcted myocardium. For histological examination, the heart tissues were fixed in 4% PFA for 24 h and then paraffin‐embedded, followed by 5 µm sectioning. The sections were deparaffinized and underwent H&E and Masson's Trichrome staining as per instructions. For WGA immunofluorescence, the sections underwent EDTA buffer antigen retrieval (pH 8.0) and were stained with WGA FITC (Thermo Fisher, Waltham, MA, USA). DAPI was used as a counterstain. The sections were imaged with LSCM, and the images were quantified with ImageJ software.

### 5.23 Statistical Analysis

GraphPad Prism 8.0 and IBM SPSS 27.0 were used for data analysis. Normality was tested prior to analysis. Normally distributed continuous data are shown as mean ± SD; non‑normally distributed data as median (P25‐P75). Categorical data are shown as counts (%). Each experiment was repeated ≥3 times. Two‑group comparisons used an unpaired Student's *t*‑test; multiple groups were compared by one‑way or two‑way ANOVA with appropriate post hoc tests. ROC curves were generated to evaluate the diagnostic performance of piR‐1873 and conventional clinical biomarkers. The DeLong test was applied to compare the difference in AUC. Significance levels: ^*^ *p* < 0.05, ^**^ *p* < 0.01, ^***^ *p* < 0.001, ^****^ *p* < 0.0001.

## Author Contributions

YZ: Methodology, Software, Writing – original draft. HQ: Conceptualization, Methodology, Software, Investigation, Resources, Writing – review and editing. YY: Conceptualization, Source. FW: Resources. TH: Visualization, Investigation. YL: Visualization, Investigation. XL: Data curation, Writing – original draft. WZ: Conceptualization, Writing – review & editing. HQ: Conceptualization, Writing – review & editing. TY: Conceptualization, Writing – review & editing, Supervision. PS: Writing – review & editing, Supervision.

## Conflicts of Interest

The authors declare no conflicts of interest.

## AI Declaration

No artificial intelligence (AI) tools were used in the preparation, generation, or modification of the figures and graphical illustrations in this manuscript.

## Supporting information




**Supporting File**: advs78282‐sup‐0001‐SuppMat.docx.

## Data Availability

The data that support the findings of this study are available from the corresponding author upon reasonable request.
