# Supplementary material for: Ultrasound‐Triggered piRNA Delivery Using Biomimetic Phase‐Changeable Nanoparticles for Theranostics of Myocardial Infarction
Source: Adv Sci (Weinh). 2026 Oct 14:e78282. Online ahead of print. doi: 10.1002/advs.78282 (PMC13669227; doi:10.1002/advs.78282)
Supplement: Supplementary file 1 [file ADVS-9999-e78282-s001.docx]

**Supporting Information**

| **Table S1:** Baseline characteristics of the study participants | | | | |
| --- | --- | --- | --- | --- |
| Variables | CON (n=30) | MI (n=30) | t/χ^2^/z值 | *P*值 |
| Age (years) | 61.97±10.25 | 68.23±14.47 | -1.936 | 0.058 |
| Male (%) | 15 (50.00) | 16 (53.33) | 0.067 | 0.796 |
| Height (m) | 1.68±0.06 | 1.66±0.08 | 0.809 | 0.422 |
| Body weight (kg) | 72.52±10.88 | 67.62±11.59 | 1.689 | 0.097 |
| BMI (kg/m2) | 25.76±2.93 | 24.41±3.02 | 1.789 | 0.079 |
| SBP (mmHg) | 124.73±8.43 | 128.77±12.57 | -1.459 | 0.151 |
| DBP (mmHg) | 76.00±7.74 | 75.80±10.43 | 0.084 | 0.933 |
| HR (bpm) | 73.93±10.88 | 76.13±11.44 | -0.763 | 0.448 |
| Current smoking (%) | 1 (3.33) | 6 (20.00) | 2.588 | 0.108 |
| Current drinking (%) | 1 (3.33) | 4 (13.33) | 0.873 | 0.350 |
| CRP (mg/L) | 0.60 (0.50-1.64) | 5.07 (3.88-15.24) | -5.782 | <0.001 |
| NT-pro-BNP (pg/mL) | 28.15 (23.04-33.46) | 1427.00 (662.25-4340.75) | -6.653 | <0.001 |
| CK-MB (U/L) | 12.00 (11.00-15.00) | 12.50 (11.00-13.25) | -0.143 | 0.887 |
| cTnI (ng/mL) | 0.01 (0.01-0.01) | 0.12 (0.03-2.62) | -6.322 | <0.001 |
| TG (mmol/L) | 1.01±0.34 | 1.51±0.47 | -4.682 | <0.001 |
| TC (mmol/L) | 4.93 (4.64-5.17) | 3.93 (3.35-4.10) | -5.841 | <0.001 |
| HDL-c (mmol/L) | 1.60±0.15 | 1.13±0.19 | 10.664 | <0.001 |
| LDL-c (mmol/L) | 2.49 (2.01-2.85) | 2.20 (1.55-2.80) | -1.042 | 0.297 |
| eGFR | 101.94 (95.74-110.52) | 81.16 (66.57-90.47) | -5.978 | <0.001 |
| LVEF (%) | 66.00 (64.00-66.00) | 50.00 (43.50-55.00) | -6.475 | <0.001 |
| piRNA | 1.02 (0.70-1.40) | 0.50 (0.46-0.87) | -4.506 | <0.001 |
| Abbreviations: MI, myocardial infarction; BMI, body mass index; SBP, systolic blood pressure; DBP, diastolic blood pressure; HR, heart rate; CRP, C-reactive protein; NT-pro-BNP, N-terminal pro-brain natriuretic peptide; CK-MB, creatine kinase MB isoenzyme; cTnI, cardiac troponin I; TG, triglyceride; TC, total cholesterol; HDL-c, high-density lipoprotein cholesterol; LDL-c, low-density lipoprotein cholesterol; eGFR, estimated glomerular filtration rate; LVEF, left ventricular ejection fraction. | | | | |

**Table S2:** Diagnostic performance of piR-1873 and conventional biomarkers for MI detection.

| Model | AUC | 95% CI | *P* value | DeLong comparison |
| --- | --- | --- | --- | --- |
| NT-pro-BNP | 1.000 | 1.000–1.000 | <0.0001 |  |
| cTnI | 0.9717 | 0.9361–1.000 | <0.0001 |  |
| piR-1873 | 0.8389 | 0.7409–0.9369 | <0.0001 |  |
| cTnI + piR-1873 | 0.9767 | 0.9419–1.000 | <0.0001 | cTnI vs cTnI + piR-1873:  *P* = 0.442 |
| NT-pro-BNP + piR-1873 | 1.000 | 1.000–1.000 | <0.0001 | NT-pro-BNP vs NT-pro-BNP + piR-1873: *P* = 1.0 |
| CRP | 0.9328 | 0.8656–0.9999 | <0.0001 |  |
| LVEF | 0.9850 | 0.9549–1.000 | <0.0001 |  |

**Table S3:** piRNA mimic and inhibitor used in this study.

| Name | Sequence (5´-3´) |
| --- | --- |
| piRNA-1873 mimic | Sense: GGC CAC ACA GAU UCA UUG CAG CAU CCU UC  Antisense: AGG AUG CUG CAA UGA AUC UGU GUG GCC UU |
| NC mimic | Sense: UUC UCC GAA CGU GUC ACG UTT  Antisense: ACG UGA CAC GUU CGG AGA ATT |
| piRNA-1873 inhibitor | GAA GGA GGA AGA AGG GG |
| NC inhibitor | CAG UAC UUU UGU GUA GUA CAA |

**Table S4:** Gene-specific primers for RT-qPCR measurements used in this study.

|  | Forward (5´-3´) | Reverse (5´-3´) |
| --- | --- | --- |
| hum-GAPDH | GTCTCCTCTGACTTCAACAGCG | ACCACCCTGTTGCTGTAGCCAA |
| mou-GAPDH | AGGTCGGTGTGAACGGATTTG | TGTAGACCATGTAGTTGAGGTCA |
| piR-23619 | GGAGTTTAATAGGTACTAAGTGGAA | NA |
| piR-28374 | TTCCATGAGACGCAGCGG | NA |
| piR-9010 | CTCAACATTCAGGGTGGTTTACA | NA |
| piR-28646 | CTGCCTTCCAAATGTTAAAATCTTGC | NA |
| piR-324 | CCTCTTAATAGTGCTACTTCCCAA | NA |
| piR-1872 | CCACACACATTCCTAGATGAAGCAA | NA |
| piR-1873 | GATTCATTGCAGCATCCTTCAAA | NA |
| mou-Usp2 | TACAGGCGAATGGCACACTT | TACATACGGGAGGGTGGACT |
| mou-Elmo1 | AGGAGGTGCTTGAACTTGCTT | GCGTGTCAGGTCACTCATCA |
| mou-Hs3st3a1 | CTGGCCTTACTTCTGGACGA | TTGTGGTAGCTGCGGTCAA |
| mou-Sema3g | TGGCTTGACTGAACCTGAAGA | GAGAGCCCACATACAGCGTT |
| mou-Ngfr | ATCTTGGCTGCTGTGGTTGT | TGTCGCTGTGCAGTTTCTCT |
| mou-Wnt7b | GTGGATGCCCGTGAGATCAA | ACATTCCAGCTTCATGCGGT |
| mou-Atp2a3 | CTCGGTCATCTGCTCTGACAA | GCTTCAGCCACCACGAACAT |

**Table S5:** Detailed information on key antibodies used in this study.

| Antibodies | Manufacturer | Catalog No. |
| --- | --- | --- |
| β-actin | ABclonal | AC026 |
| p-RIP1 | Zenbio | R10014 |
| RIP1 | ABclonal | A7414 |
| p-RIP3 | Abcam | AB222320 |
| RIP3 | ABclonal | A5431 |
| p-MLKL | Affinity | AF7420 |
| MLKL | Boster | M00535-1 |
| USP2 | Proteintech | 10392-1-AP |
| ubiquitin | Proteintech | 80992-1-RR |
| N-cadherin (Extracellular) | ThermoFisher | PA5-85341 |
| N-cadherin (Intracellular) | ThermoFisher | MA1-159 |

**Table S6:** FISH and Pull down probe sequence used in this study.

| Probe name | Probe sequence (5´-3´) |
| --- | --- |
| FISH-piRNA-1873 | GAAGGATGCTGCAATGAATCTGTGTGGCC |
| FISH-NC | TGCTTTGCACGGTAACGCCTGTTTT |
| Pull down-piRNA-1873 | GGCCACACAGAUUCAUUGCAGCAUCCUUC |
| Pull down-NC | AACGATTTCCGGCTCCATCCCGTGCATAA |

**Table S7:** Quantification of PFH content and apparent PFH retention efficiency determined by ^19^F-NMR analysis.

| Parameter | Value |
| --- | --- |
| TFA internal standard | 10 μL, 99.9% |
| TFA amount | 130.461 μmol |
| PFH/TFA molar ratio | 0.107 |
| PFH amount in 10 μL aliquot | 13.959 μmol (4.719 mg) |
| Total PFH content in final suspension | 47.188 mg |
| PFH concentration in final suspension | 471.88 mg/mL |
| Initial PFH input | 83.45 mg |
| Apparent PFH retention efficiency | 56.55% |

**Figure S1.** A mouse MI model was established using left anterior descending (LAD) ligation. A) Left ventricular long-axis end-diastolic 2D and M mode echocardiography 28 days after LAD ligation in sham and MI mice. B) Quantification of left ventricular ejection fraction (LVEF), left ventricular fractional shortening (LVFS), left ventricular end-diastolic diameter (LVDd), and left ventricular end-systolic diameter (LVDs) (n = 7). C) Quantification of fibrosis area (n=7). D) Relative expression levels of up-regulated piRNAs in mouse myocardial tissue were assessed by RT-qPCR (n = 7). E) Relative expression levels of up-regulated piRNAs in H_2_O_2_-treated cardiomyocytes were assessed by RT-qPCR (n = 3). **p* < 0.05, ***p* < 0.01, *****p* < 0.0001 compared with indicated groups.

**Figure S2.** Cardiomyocyte necroptosis model was induced by H_2_O_2_. A) RT-qPCR was used to detect the relative expression level of piR-1873 in neonatal ventricular cardiomyocytes (NVCM) and neonatal cardiac fibroblasts (NCF) (n = 3). B) HL-1 cells were treated with H_2_O_2_ or hypoxia, and RT-qPCR was used to detect of the relative expression levels of piR-1873 under different treatment conditions and time points (n = 3). C,D) PI staining was used to assess necrosis of HL-1 cardiomyocytes induced by different concentrations of H_2_O_2_, and quantification of the proportion of PI-positive cells (n = 3). Scale bars: 100 μm. E,F) PI staining was used to assess necrosis of HL-1 cardiomyocytes induced by H_2_O_2_ at the concentration of 600 μM for different treatment times, and quantification of the proportion of PI-positive cells (n = 3). Scale bars: 100 μm. **p* < 0.05, ***p* < 0.01, ****p* < 0.001, *****p* < 0.0001 compared with indicated groups.

**Figure S3.** Temporal dynamics analysis of circulating piR-1873 and classical cardiac injury biomarkers after MI. A-C) Representative temporal profiles of serum piR-1873 (A), cTnI (B), and CK-MB (C) levels at different time points after LAD ligation. Each measurement was performed in technical triplicates from a single mouse.

**Figure S4.** piR-1873 overexpression or knockdown does not affect HL-1 cells proliferation or apoptosis. A) RT-qPCR was used to detect the overexpression efficiency of piR-1873 mimic in HL-1 cells (n = 3). B) RT-qPCR was used to detect the knockdown efficiency of the piR-1873 inhibitor in HL-1 cells (n=3). C,D) CCK-8 assay was used to detect the effect of piR-1873 overexpression and knockdown on the proliferation of HL-1 cells (n = 3). E,F) Edu staining was used to detect the effect of piR-1873 overexpression or knockdown on the proliferation of HL-1 cells, and to quantify the proportion of Edu-positive cells (n = 3). Scale bars: 100 μm. G,H) Tunel staining was used to determine the effect of overexpression or knockdown of piR-1873 on the proportion of apoptotic HL-1 cells, and to quantify the proportion of Tunel-positive cells (n = 3). Scale bars: 100 μm. ns: no significance, **p* < 0.05, ****p* < 0.001 compared with indicated groups.

**Figure S5.** Dispersion behavior in different media and *in vitro* cytocompatibility of piR-NPs. A) Photographs of piR-NPs dispersed in PBS, 0.9% saline, DMEM, and DMEM medium containing 15% FBS. B) HL-1 cells pretreated with H_2_O_2_ for 8 h, then incubated with nanoparticles for 12, 24, 36, or 48 h; proliferation assessed by CCK-8 (n = 3). ns: not significance compared with indicated groups.

**Figure S6.** Characterization of piRNA loading, membrane coating properties, and membrane protein topology of piR-NPs. A) Determination of piRNA encapsulation efficiency in three independent batches of piR-NPs. B) Quantification of piRNA loading capacity in three independent batches of piR-NPs (μg piRNA/mg PEI-PLA). C) Quantitative ^19^F NMR spectrum of the final PFH-containing nanoparticle suspension using TFA as the internal standard. The inset shows the TFA CF3 signal at δF = −75.91 ppm (I = 3.000) and the PFH terminal CF3 region at δF ≈ −80.1 to −81.3 ppm (I = 0.642) used for quantitative estimation. D) Determination of PFH retention efficiency in three independent batches of piR-NPs. E) Quantification of the membrane protein-to-polymer core mass ratio (w/w) in piR-NPs by BCA assay. F) Evaluation of the membrane coating efficiency of piR-NPs using the FITC fluorescence quenching assay. G,H) Proteinase K protection assay evaluating the exposure and topology of membrane proteins on the surface of piR-NPs. N-cadherin was used as a membrane protein marker, and extracellular and intracellular domains were detected by Western blotting followed by quantitative analysis (n = 3). ns: no significance, ***p* < 0.01, ****p* < 0.001, *****p* < 0.0001 compared with indicated groups.

**Figure S7.** Ultrasound responsiveness of piR-NPs and ultrasound imaging-guided monitoring of cardiac function after MI. A,B) Representative B-mode and contrast-enhanced ultrasound (CEUS) images of piR-NPs after stimulation with different ultrasound intensities (0, 0.5, 1.0, 1.5, and 2.0 W/cm^2^), followed by quantitative analysis of CEUS echo intensity (n = 3). C,D) Representative B-mode, CEUS, zoomed CEUS, and M-mode echocardiographic images of mice injected with piR-NPs followed by ultrasound triggering at different time points after LAD ligation (0, 1, 3, 5, 7, 14, and 28 days). CEUS echo intensity and LVEF were quantitatively analyzed. ns: no significance, ****p* < 0.001 compared with indicated groups.

**Figure S8.** Effects of ultrasound and commercial transfection reagent-mediated piR-1873 delivery on cardiac function, myocardial structure, and biosafety in mice with MI. A) Representative left ventricular long-axis end-diastolic 2D and M-mode echocardiography images of mice in different groups at 28 days after MI. B) Quantitative analysis of left ventricular structural and functional parameters, including LVEF, LVFS, LVDd, and LVDs (n = 7). C,E) Representative hematoxylin and eosin (H&E) and Masson staining images of cardiac tissues from different groups, with quantitative analysis of myocardial fibrosis area (n = 7), scale bar: 500 μm. D,F) Representative wheat germ agglutinin (WGA) fluorescence staining images of cardiac tissues for evaluation of cardiomyocyte cross-sectional area, with quantitative analysis of cardiomyocyte area (n = 7), scale bar: 100 μm. G) Representative H&E staining images of major organs, including the liver, spleen, lung, and kidney, for evaluation of *in vivo* histological safety (n = 7), scale bar: 200 μm. H) Serum biochemical analysis, including alanine aminotransferase (ALT), aspartate aminotransferase (AST), blood urea nitrogen (BUN), creatinine (CREA), triglyceride (TG), and total cholesterol (CHO), for evaluation of systemic biosafety (n = 7). ns: no significance, ***p* < 0.01, *****p* < 0.0001 compared with indicated groups.

**Figure S9.** Validation of U6 and GAPDH stability as reference transcripts for RT-qPCR normalization. A) Raw Ct values of U6 under different experimental conditions (n = 3). B) Raw Ct values of GAPDH under different mimic/inhibitor treatments (n = 3). ns, no significant difference among the indicated groups.
